# Parallel Alpine Differentiation in *Arabidopsis arenosa*

**DOI:** 10.3389/fpls.2020.561526

**Published:** 2020-12-08

**Authors:** Adam Knotek, Veronika Konečná, Guillaume Wos, Doubravka Požárová, Gabriela Šrámková, Magdalena Bohutínská, Vojtěch Zeisek, Karol Marhold, Filip Kolář

**Affiliations:** ^1^Department of Botany, Charles University, Prague, Czechia; ^2^Institute of Botany, The Czech Academy of Sciences, Průhonice, Czechia; ^3^Institute of Botany, Slovak Academy of Sciences, Bratislava, Slovakia; ^4^Department of Botany, University of Innsbruck, Innsbruck, Austria

**Keywords:** adaptation, alpine environments, *Arabidopsis*, convergence, parallel evolution, phenotypic parallelism

## Abstract

Parallel evolution provides powerful natural experiments for studying repeatability of evolution and genomic basis of adaptation. Well-documented examples from plants are, however, still rare, as are inquiries of mechanisms driving convergence in some traits while divergence in others. *Arabidopsis arenosa*, a predominantly foothill species with scattered morphologically distinct alpine occurrences is a promising candidate. Yet, the hypothesis of parallelism remained untested. We sampled foothill and alpine populations in all regions known to harbor the alpine ecotype and used SNP genotyping to test for repeated alpine colonization. Then, we combined field surveys and a common garden experiment to quantify phenotypic parallelism. Genetic clustering by region but not elevation and coalescent simulations demonstrated parallel origin of alpine ecotype in four mountain regions. Alpine populations exhibited parallelism in height and floral traits which persisted after two generations in cultivation. In contrast, leaf traits were distinctive only in certain region(s), reflecting a mixture of plasticity and genetically determined non-parallelism. We demonstrate varying degrees and causes of parallelism and non-parallelism across populations and traits within a plant species. Parallel divergence along a sharp elevation gradient makes *A. arenosa* a promising candidate for studying genomic basis of adaptation.

## Introduction

When and why does evolution result in a predictable outcome remain challenging questions in evolutionary biology. Parallel emergence of identical phenotypes in similar environments brings one of the most intuitive examples of the action of natural selection and provides independent biological replicates allowing for identifying the genetic basis and phenotypic outcomes of adaptation (Elmer and Meyer, 2011; Losos, 2011). In particular, parallel evolution of genetically determined phenotypically distinct entities within a species, so-called ecotypes, provide valuable insights into processes of local adaptation and incipient ecological speciation in nature (Bolnick et al., 2018; Thompson et al., 2019). In contrast to well-studied examples of ecotypic parallelism from animals such as fishes (Rundle et al., 2000; Jones et al., 2012; Reid et al., 2016; Stuart et al., 2017), snails (Butlin et al., 2014; Ravinet et al., 2016), and stick insects (Nosil et al., 2002; Soria-Carrasco et al., 2014), mechanisms driving parallel evolution in plants are largely unknown (but see Roda et al., 2013b; Bertel et al., 2018).

While directional selection driven by similar environment shall lead to convergence, alternative neutral and selective forces may counteract it, leading to larger genetic and phenotypic divergence among the derived ecotypes (reviewed in Bolnick et al., 2018). Specifically, both stochastic processes such as genetic drift, varying intensity of gene flow and different pools of initial variation available for selection as well as deterministic processes such as selection in response to locally distinct micro-environments and genetic architecture of the traits can lead to non-parallel patterns in phenotypic variation (Elmer et al., 2014; Langerhans, 2018). Consequently, there is no clear-cut border between parallel and non-parallel phenotypes, and the extent of parallelism may vary over traits or particular sets of compared populations (Stuart et al., 2017). Finally, apparent similarity across populations can reflect short-term environmentally induced traits (phenotypic plasticity), however, the relative contribution of the heritable vs plastic forces in the manifestation of phenotypic parallelism remains unknown (Pfennig et al., 2010).

Multiple (*N* > 2) independent environmental transitions within a species provide powerful naturally replicated systems to infer the mechanisms driving parallel phenotypic evolution as well as the extent of variation in parallel vs non-parallel response. Such systems allow multiple pairwise comparisons, in contrast to systems comparing only two parallel transitions. Indeed, independently evolved animal ecotypes documented that a combination of both stochastic and deterministic processes led to a varying extent of parallelism (Bolnick et al., 2018). In contrast, such detailed inquiries in plants are scarce due to paucity of well-established systems that involve multiple replicates of environmental transitions within a species. The limited evidence from the so far investigated systems, *Heliosperma pusillum* (Trucchi et al., 2017; *Caryophyllaceae*; five pairs of alpine-foothill ecotypes, Bertel et al., 2018) and *Senecio lautus* (Roda et al., 2013a, *Asteraceae*; seven pairs of dune-heathland ecotypes, Roda et al., 2013b), suggest considerable morphological divergence among the independently formed ecotypes despite similar environmental triggers, perhaps as a result of genetic drift (Trucchi et al., 2017) or gene flow (Roda et al., 2013a). However, to infer mechanisms driving parallel evolution in plants in general and its genetic basis in particular, additional genetically well-tractable plant systems are needed.

Alpine populations of *Arabidopsis arenosa* represent a promising system for addressing drivers and consequences of parallel evolution in a genomic and molecular genetic context of the well-researched *Arabidopsis* genus. The species thrives mostly in low to mid-elevations (up to ∼1,000 m a.s.l.) of Central and Eastern Europe, but scattered occurrences in treeless alpine habitats (∼1,500–2,500 m a.s.l.) have been described from four mountain regions in the floristic literature (Melzer, 1960; Měsíček and Goliašová, 2002; Bartok et al., 2016). Alpine environments are generally well-poised for inquiry of plant parallel adaptation. On one hand, high elevations pose a spectrum of challenges to plant life, potentially triggering directional selection, such as freezing and fluctuating temperatures, strong winds, increased UV radiation and low partial pressure of carbon dioxide. Such pressures are believed to jointly trigger emergence of distinct alpine phenotypic “syndromes” (e.g., contracted rosette plants, dense cushions, and giant rosettes) that have been recurrently formed in distinct mountain ranges throughout the world (Hedberg and Hedberg, 1979; Körner, 2003; Halbritter et al., 2018; Konečná et al., 2019). Indeed, alpine *A. arenosa* constitutively exhibits a distinct morphotype characterized by lower stature, less-lobed and thicker leaves, larger flowers and wider siliques (Měsíček and Goliašová, 2002). On the other hand, the island-like distribution of alpine habitats promotes parallel colonization of individual alpine “islands” by the spatially closest foothill populations (Levin, 2001). In line with this, a recent genomic study of *A. arenosa* (Monnahan et al., 2019) demonstrated geographical structuring of its range-wide genetic diversity, suggesting that the alpine ecotype might be of a polytopic origin. This hypothesis, as well as the extent of phenotypic parallelism and its plastic vs. genetic basis, however, remains untested.

In this study, we sampled multiple alpine and adjacent foothill *A. arenosa* populations covering all mountain regions known to harbor the alpine ecotype. Using genome-wide SNP genotyping we tested our main hypothesis that alpine environment within each mountain region has been colonized independently (parallel origin scenario) as opposed to clustering of the alpine populations together (single origin scenario). Indeed, a combination of genetic structure analyses and modeling revealed multi-parallel origin of the alpine populations. Thus, we combined analyses of field-sampled phenotypic data and a common garden experiment to assess whether the independent alpine transitions are also associated with phenotypic parallelism. Specifically, we ask: (1) Had independent alpine colonization triggered similar phenotypic transitions in distinct mountain regions? (2) Does the alpine phenotype remain divergent from the “typical” foothill form in standardized conditions and, if so, which characters contribute to the genetically determined alpine syndrome? (3) In contrast, are there traits exhibiting a rather opposite, non-parallel response?

## Materials and Methods

### Field Sampling

*Arabidopsis arenosa* is a perennial outcrosser encompassing diploid and autotetraploid populations (Kolář et al., 2016b; Monnahan et al., 2019) that is widespread in foothill (colline to sub-montane) elevations across Central and Eastern Europe. Scattered occurrences of morphologically distinct populations have been recorded from four distinct mountain regions in Europe, sometimes treated as a separate species “*A. neglecta*”: Eastern Alps (Melzer, 1960), Eastern (Pachschwöll and Pachschwöll, 2019), Southern (Bartok et al., 2016), and Western Carpathians (Měsíček and Goliašová, 2002). While only tetraploid populations colonized the alpine stands in the former three regions, both diploid and tetraploid populations reached the alpine belt in the Western Carpathians. As the two cytotypes still occupy distinct alpine sub-regions there (diploids in Vysoké Tatry Mts. and tetraploids in Západné Tatry Mts.; Wos et al., 2019), we kept the ploidies as separate units for the sake of clarity. In sum, we hereafter refer to five regions: Niedere Tauern and surrounding foothills of the Eastern Alps (NT region, tetraploid), Rodna Mts. and adjacent regions of Eastern Carpathians (RD, tetraploid), Fǎgǎraş Mts. in Southern Carpathians (FG, tetraploid), Vysoké Tatry Mts. and adjacent foothill diploid populations in Western Carpathians (VT, diploid), Západné Tatry Mts. and adjacent foothill tetraploid populations in Western Carpathians (ZT, tetraploid).

In each mountain region we sampled multiple populations from foothill habitats (semi-shaded rocky outcrops, screes and steep slopes; “foothill ecotype”) and from alpine sites (screes and rocky outcrops above the timberline; “alpine ecotype”). As this heliophilous species occurs on “islands” of environmentally suitable conditions (rocky outcrops in lower elevations, rocks and screes in isolated glacial cirques in the alpine zone), the population was defined as a set of individuals occurring in a spatially discrete area in a homogeneous vegetation type surrounded by habitats with unsuitable conditions for the species (e.g., forests, dense grasslands, and arable land). Both ecotypes are separated by a clear distribution gap spanning at least 500 m of elevation which also corresponds with the timberline. We avoided sampling plants in riverbeds immediately below the alpine populations that could represent recent colonizers germinated from washed seeds of the originally alpine plants. We sampled adult individuals from a total of 58 populations – 30 from foothill and 28 from alpine habitats. Within each population, we collected on average 17 individuals at the full-flowering stage: a small part of fresh tissue for ploidy determination, leaf tissue desiccated in silica gel for genotyping and vouchers for morphometrics. We selected largest rosette leaf, second stem leaf from the base and one random flower from the terminal inflorescence and fixed them onto paper with transparent tape for detailed measurements of organ sizes; the entire individual was then press-dried. For each population we also sampled the following local environmental parameters at a microsite with abundant occurrence of *A. arenosa*: (i) vegetation samples (phytosociological relevés, each covering an area of 3 × 3 m) recording percentage of the area covered by herb layer and listing all vascular plant species and (ii) mixed rhizosphere soil samples from five microsites within the vegetation sample.

Ploidy level of each sampled individual was determined using flow cytometry as described by Kolář et al. (2016b).

### Experimental Cultivation

In addition to field sampling, we established a common garden experiment to test whether the plants of alpine origin keep their distinct appearance when cultivated in the foothill-like conditions. We used seeds of *A. arenosa* collected from 16 natural populations overlapping with those sampled for field phenotyping and genotyping (except for pop. AA254 that was used in the experiment as a replacement of spatially close, <10 km, pop. AA145 which was included in the field dataset). The populations represent four regions (NT, VT, ZT, and FG) and two distinct elevations (two foothill and two alpine populations per region; see Supplementary Table S1 for locality details). To minimize maternal effect of the original localities, we firstly raised one generation in growth chambers under constant conditions. To simulate outcrossing in natural populations, each plant was hand-pollinated by a mixture of pollen from the same population (∼14 individuals) for a period of one week; the offspring thus represented a genetically variable mixture of full- and half-sibs. The plants used for phenotyping were raised in the Botanical Garden of the University of Innsbruck (Austria) situated in the Alpine valley, i.e., in conditions resembling the foothill habitat. Phenotypic traits were collected on all plants in the full-flowering stage in an identical way as for the field sampling. For details on cultivation conditions, see Supplementary Methods S1.

### Inference of Parallel Origins

We genotyped 156 individuals from 46 populations (2–8 individuals per population, 3.5 on average) using the double-digest RADseq protocol of Arnold et al. (2015). For an additional 44 individuals from 11 populations genome-wide SNP data were already available from a genome resequencing study (Monnahan et al., 2019). Raw reads processing and filtration generally followed our earlier study (31); for details see the Supplementary Methods S1.

We inferred population grouping using several complementary approaches. Firstly, we ran *K*-means clustering, a non-parametric method with no assumption on ploidy, in adegenet v1.4-2 using 1000 random starts for each *K* between 1 and 20 and selected the partition with the lowest Bayesian Information Criterion (BIC) value (Jombart, 2008). Secondly, we used model-based method FastStructure (Raj et al., 2014). We randomly sampled two alleles per tetraploid individual (using a custom script) – this approach has been demonstrated not to lead to biased clustering in autotetraploid samples in general (Stift et al., 2019) and *Arabidopsis* in particular (Monnahan et al., 2019). We ran the FastStructure with 10 replicates under *K* = 5 (the same number as for *K*-means clustering, representing the number of the regions) and additionally only for tetraploid individuals under *K* = 4. Finally, we displayed genetic distances among individuals using principal component analysis (PCA) as implemented in adegenet v.2.1.1. For clustering analyses, we used random thinning over 150 kb windows (length of our RAD-locus) to reduce the effect of linkage and removed singletons resulting in a dataset of 4,341 SNPs. PCA, AMOVA and genetic distances (see below) were calculated using the full set of 103,928 filtered SNPs.

**FIGURE 1:**
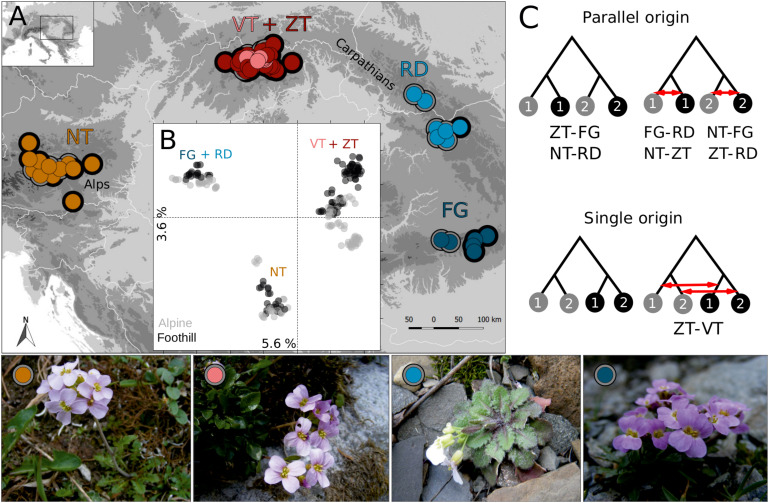
Parallel alpine differentiation of *Arabidopsis arenosa* in Central Europe inferred from genome-wide SNP variation. **(A)** Geographic distribution of sampled populations colored by *K*-means clusters (circles with different colors) and classified according to the ecotype (black rim = foothill, gray rim = alpine). **(B)** Principal component analysis of the individuals colored according to ecotype and labeled according to region (color corresponding to panel **A**). **(C)** Four scenarios addressed by coalescent simulations; gray dots = alpine populations, black dots = foothill populations, numbers = regions, red arrows = migration events. The scenario with best fit to the observed allele frequency spectra for each pair of regions tested is indicated by its respective code below the scheme. Illustrative photos of convergent alpine *A. arenosa* ecotypes from four regions are presented at the bottom. Note that the VT region is occupied by diploids while all other regions are represented by autotetraploid populations.

Additionally, we tested for parallel origin of alpine populations using coalescent simulations in *fastsimcoal v.26* (Excoffier et al., 2013) – an approach that is also suitable for autotetraploids and mixed-ploidy systems (Arnold et al., 2012). We constructed unfolded (polarization following Monnahan et al., 2019) four-dimensional joint allele frequency spectra from genome-wide SNPs from a subset of sufficiently sampled populations, one alpine and one foothill per region (∼ 176–417 k SNPs per population, see Supplementary Table S2 and Supplementary Methods S3 for details; one-dimensional AFS of the populations are published in Monnahan et al., 2019). We compared all regions occupied by tetraploid populations (NT, RD, FG, and ZT) in a pairwise manner. Taking into account single origin of the widespread *A. arenosa* tetraploid cytotype (Arnold et al., 2015; Monnahan et al., 2019), we had not compared all diploid-tetraploid pairs but only the spatially closest diploid and tetraploid populations from Western Carpathians (VT and ZT) for which a complex reticulation relationships was suggested previously (Wos et al., 2019). Briefly, for each pair of regions, we compared the fit of our data with two competing topologies: (i) sister position of alpine and foothill populations from the same region (i.e., parallel origin) and (ii) sister position of populations from distinct regions but belonging to the same ecotype (i.e., single origin of each ecotype). For each topology we either assumed or not assumed secondary contact (between-ecotype gene flow) within each region, what resulted in a total of four scenarios per regional pair (Figure 1C). As the main aim of the study was testing for single vs parallel origin of the ecotypes (i.e., model comparison) we had not followed with additional analyses quantifying population divergence (i.e., parameter estimates) within each region.

We used hierarchical analysis of molecular variance (AMOVA) implemented in the R package pegas to test for genetic differentiation (i) among regions and among populations within regions and (ii) among ecotypes and among populations within each ecotype (both in the total dataset and in each region separately). We calculated nucleotide diversity (π) and Tajima’s D for each population with at least four individuals (*N* = 26) and pairwise differentiation (F_*ST*_) between these populations using custom python3 scripts (available at https://github.com/mbohutinska/ScanTools_ProtEvol).

### Ecological Data

To assess environmental differences among the populations, we combined broad-scale climatic parameters acquired from a database (SolarGIS, version 1.9, operated by GeoModel Solar, Bratislava, Slovakia) with local conditions sampled at the original site of each population. First, we estimated the average values of three climatic variables: Precipitation, Temperature and Photosynthetic Active Radiation (PAR), over April, May, and June, which correspond to the main growth period of *A. arenosa*. Second, we measured soil pH of each site by thermo-corrected electrode (WTW Multilab 540, Ionalyzer, pH/mV meter) at the Analytical Laboratory of the Institute of Botany (Průhonice, Czech Republic). Finally, we inferred local environmental conditions from the vegetation samples: (i) total vegetation cover of herb layer and (ii) Ellenberg Indicators Values (EIVs) calculated from the species list using JUICE (Tichý, 2002). EIVs provide estimates of environmental characteristics of the sites inferred from species composition based on expert knowledge (Ellenberg, 1992). In total, we recorded 671 plant species of which 76% have EIVs. We used only EIVs for Light, Nutrients and Moisture as the remaining EIVs (for Temperature, Continentality, and Soil Reaction) overlapped with the above-described climatic and soil variables. In sum, our dataset of environmental parameters comprised the following eight variables: Precipitation, Temperature, PAR, soil_pH, Vegetation_cover, EIV_Light, EIV_Nutrients and EIV_Moisture. No pair of the parameters exhibited >0.8 Pearson correlation (Supplementary Table S3).

### Phenotypic Traits

To test for parallel phenotypic response to alpine environment, we described the morphology of each plant sampled using 16 traits (999/223 vouchers in total scored for the field and common garden datasets, respectively). On each plant we measured 12 phenotypic characters describing the overall shape and size of both vegetative and reproductive organs (except for siliques which were not fully developed in the full-flowering stage which our sampling aimed at). To assess variation in shape of the organs independent of absolute size, we further derived four ratios (all characters are described and listed in Supplementary Table S4). Missing values in the field dataset (1.5% in total) were replaced by population means. For statistical analyses all characters except four (PL, PW, SL and SW), were log-transformed to approach normal distribution. No pair of traits was very strongly correlated (>0.8, Supplementary Table S3).

### Statistical Analyses of Ecological and Morphological Data

We used PCA calculated by base R function *prcomp* to visualize ecological differences among populations and morphological differences among individuals. Separate PCAs have been calculated on standardized (zero mean and unit variance) sets of (i) the eight climatic and local environmental variables and the 16 morphological traits recorded on the individuals collected (ii) in field and (iii) in a common garden experiment. Overall differentiation in environmental conditions and morphology of the ecotypes were tested by permutation multivariate analyses of variance (permanova). We first calculated Euclidean distances among population (environmental data) or individual (field and common garden morphological data) values using *dist* function and then ran a permanova test with *adonis2* function (number of permutations = 30,000) in R package vegan 2.5–4. In addition, we quantified the range of morphological variation of each ecotype by calculating disparity as the median distance between each individual and centroid of their corresponding ecotype in the ordination space using the R package dispRity.

To quantify morphological differentiation between pre-defined groups (ecotypes and regions) across all traits we ran classificatory discriminant analysis with cross-validation as implemented in Morphotools 1.1 (Koutecký, 2014). To assess relative contribution of individual morphological characters to the between foothill-alpine differentiation individuals we calculated a constrained ordination (linear discriminant analysis, LDA) in Morphotools. We calculated the discriminant analyses and permanova tests (i) for complete datasets and then (ii) separately for each region with ecotype as a factor to assess major drivers of foothill-alpine phenotypic differentiation within each region and (iii) separately for each ecotype with region as a factor to quantify variation in between-region phenotypic differentiation within each ecotype.

Finally, we ran generalized linear mixed models (GLM) for each of the 16 phenotypic traits to test for the effects of ecotype, region and their interaction (individual-based data with population as a random factor, *lme* function) in nlme package v3.1-137. Parallelism in foothill-alpine differentiation was considered for traits with significant effect of ecotype but non-significant ecotype × region interaction (lack of regionally specific differences between ecotypes) that was revealed consistently in both field and common garden datasets. In turn, a trait with significant effect of ecotype × region interaction was indicative of non-parallelism (evidence for regional-specific differences). Finally, traits exhibiting significant ecotypic effect in the field dataset but not in common garden were considered as plastic with respect to alpine differentiation. To assess the degree of parallelism and non-parallelism in a more quantitative way, effect sizes of each factor and their interaction were estimated from a linear model using a partial eta-squared method (Etasq function) in R package heplots. Similarly as above, large ecotypic effect only was indicative of parallelism while strong contribution of the ecotype × region interaction implied non-parallel foothill-alpine differentiation in that particular trait (Stuart et al., 2017). All the above analyses were run separately for the field and common garden datasets.

## Results

### Genetic Structure and Diversity

We confirmed the presence of tetraploid populations in four mountain regions (NT, ZT, RD, and FG) while only diploids represented the Vysoké Tatry (VT) region in our sample. For the 200 RAD-sequenced individuals we gathered 103,928 SNPs of average depth 30× (1.37% missing data). Non-hierarchical K-means clustering revealed that the populations cluster under K = 5 (partition supported by the lowest Bayesian information criterion, Supplementary Figure S1) according to the geographic regions disregarding their alpine or foothill origin (i.e., the ecotype; Figure 1). Populations from the spatially close VT and ZT regions, which differed by ploidy, were the single exception: here, the majority of the alpine diploid populations (from VT region) and some alpine tetraploid (ZT) populations formed a separate cluster distinct from the remaining Western Carpathian samples, regardless of ploidy. FastStructure run under *K* = 5 supported this clustering and revealed clear separation of all groups but the VT and ZT groups, which were remarkably admixed (Supplementary Figure S2). Principal component analysis confirmed spatial, not ecotypic, clustering and revealed three main clusters: populations from the RD and FG regions were separated from the VT and ZT cluster along the first axis, while NT populations differentiated from the rest along the second axis (Figure 1B).

Coalescent simulations demonstrated that parallel origin scenarios are more likely than scenarios assuming single origin of alpine ecotype and this result was consistent across all combinations of tetraploid populations (Akaike weights supporting parallel origin ranged 0.99–1 across the pairwise comparisons of regions; Supplementary Table S5, see also Supplementary Figure S3 for the distribution of AIC values). In contrast, for the spatially close diploid (VT) and tetraploid (ZT) populations the scenario involving sister position of alpine populations of both ploidies was preferred (Akaike weight for the scenario assuming single origin followed by migration was 1; Supplementary Table S5). In summary, genetic analysis coherently demonstrated polytopic origin of the alpine ecotype in four distinct mountain ranges (Alps – NT, Eastern Carpathians – RD, Southern Carpathians – FG, and Western Carpathians – VT and ZT; Figure 1C). In order to account for the ploidy difference among the Western Carpathian populations, we kept the VT and ZT populations separate in the following analyses. Additional analyses when the VT and ZT populations were merged into a single unit (Supplementary Table S6), demonstrated that such alternative grouping does not lead to qualitatively different results with respect to the patterns in alpine-foothill differentiation.

Ecotypic differentiation explained a negligible proportion of genetic variance (3%) while the five regions accounted for 20% of variation (AMOVA analysis, see Supplementary Table S7). Consequently, genetic differentiation between foothill and alpine populations was non-significant within any of the mountain regions in separate AMOVA analyses (Table 1, see also pairwise *F*_*ST*_ among populations, Supplementary Table S8). These results suggest there is overall low inter-population divergence within each region, and such observation is consistent across regions. The alpine colonization was not accompanied by a reduction in genetic diversity, as populations belonging to both ecotypes exhibited similar nucleotide diversity overall (Wilcoxon test, *W* = 77, *p* = 0.93) as well as within each mountain region separately and also Tajima’s *D* values were close to neutrality (i.e., zero) in both ecotypes (Table 1).

**TABLE 1:** Genetic and morphological diversity and differentiation of foothill and alpine populations of *A. arenosa* from the five mountain regions sampled in Central Europe.

	Genetic diversity and differentiation					Field morphology			Common garden morphology		
	N^1^	AMOVA (%)^2^	Genetic diversity^3^	Pairwise Fst^4^	Tajima’s D^3^	N^1^	CDA^5^ (%)	Differentiation^6^	N^1^	CDA^5^ (%)	Differentiation^6^
**Grouping by region**											
All populations	200	20	0.048	0.132	−0.096	999	43	6.43***	223	47	4.00*
Foothill	109	23	0.049	0.120	−0.148	559	65	14.1***	117	62	6.58***
Alpine	91	30	0.048	0.139	−0.025	440	48	10.75***	106	75	19.94***
**Grouping by ecotype**											
All regions	200	3	0.049/0.048		−0.148/ −0.025	999	89	49.25***	223	83	108.5***
NT (Niedere Tauern)	41	n.s.	0.047/0.045	0.105/0.089	−0.022/0.069	232	100	8.83**	60	63	6.52**
VT (Vysoké Tatry)	73	n.s.	0.047/0.047	0.066/0.068	0.128/ −0.075	380	89	17.15***	48	83	60.91***
ZT (Západné Tatry)	42	n.s.	0.046/0.051	0.049/0.045	−0.251/ −0.229	204	90	16.36***	59	93	17.00***
RD (Rodna)	22	n.s.	0.058/0.060	−/–	−0.263/−0.104	85	93	3.42*	–	–	–
FG (Făgăraş)	22	n.s.	0.055/0.042	0.062/0.119	−0.173/0.225	98	100	50.96***	56	95	113.61***

^1^N RAD-sequenced/phenotyped individuals. ^2^Among-group genetic variation component (in %) as explained by hierarchical AMOVA. ^3^Pairwise nucleotide diversity (π) and Tajima’s *D* averaged over populations with ≥ 4 individuals (foothill/alpine ecotypes, respectively). ^4^Pairwise *F*_*st*_ averaged over populations with ≥ 4 individuals (foothill/alpine ecotypes, respectively). ^5^% of correct classification into ecotype/regional group as inferred by classificatory discriminant analysis of the 16 morphological characters. ^6^*F*-values and significance (*P < 0.05, ***P < 0.01, and ***P < 0.001) of permanova analysis of the 16 morphological characters. Parallel origin of alpine ecotype.

### Habitat Differentiation

**FIGURE 2:**
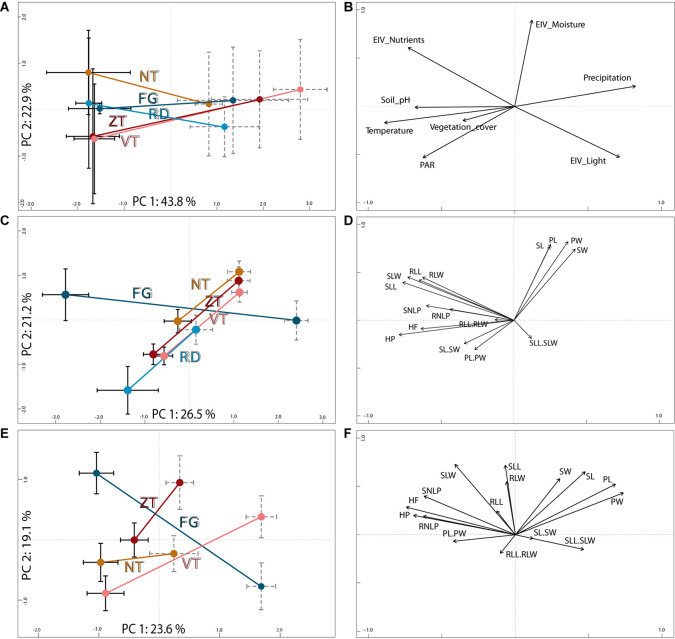
Parallel ecological **(A,B)** and field- **(C,D)** and common garden-based **(E,F)** morphological divergence of foothill and alpine populations of *A. arenosa* from the five mountain regions. The diagrams display results of principal component analyses (PCA) based on **(A)** eight environmental parameters of the original sampling sites of all populations with **(C)** 16 morphological traits collected in 58 wild populations, and **(E)** the same 16 traits sampled in a subset of 16 populations cultivated in a common garden. Centroids (dots) and standard error bars (black solid = foothill, gray dashed = alpine) are depicted for each region and ecotype; the ecotypes from the same region are connected by a line and colored as in Figure 1. The corresponding loadings of each environmental parameter **(B)** or morphological trait **(D,F)** are depicted. See Table S3 for explanation of individual codes of the traits.

Environmental conditions of foothill and alpine sites significantly differed (permutational multivariate analysis of variance of populations, permanova, *F* = 16.92, *p* < 0.001; Figures 2A,B). In contrast, populations from different mountain regions did not differ based on the environmental parameters recorded (*F* = 1.21, *p* = 0.30).

### Morphological Differentiation in Natural Populations

Field sampled alpine individuals were overall morphologically differentiated from their foothill counterparts as revealed by their separation in PCA (Figures 2C,D and Supplementary Figure S4), high (87%) classification success in the classificatory discriminant analysis and significant effect of ecotype in permanova (*F* = 49.25, *p* < 0.001). Morphological differentiation by ecotype was also significant for each region separately, with high classification success (89–100% across regions, Table 1). Overall, morphological differentiation measured by disparity was significantly higher among the foothill than among the alpine individuals (*F*_1_,_997_ = 113.4, *p* < 0.001) suggesting that alpine individuals were morphologically more similar to each other than were their foothill counterparts.

**FIGURE 3:**
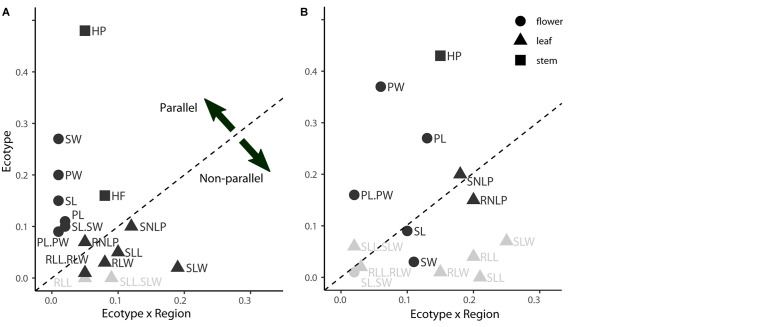
Variation in the extent of parallelism among individual phenotypic traits sampled in the field **(A)** and common garden **(B)**. The extent of parallelism was estimated as effect sizes (Eta^2^) in linear model addressing the effect of Ecotype (foothill-alpine morphological divergence), Region (divergence between mountain regions) and their interaction, calculated separately for each trait across all populations sampled. The effect size of Ecotype (*y*-axis) shows the extent to which a given trait diverges predictably between ecotypes, i.e., in parallel, while the Ecotype × Region effect size (*x*-axis) quantifies the extent to which foothill-alpine phenotypic divergence varies across regions (i.e., deviates from parallel). Points falling above the dashed line have a larger Ecotype effect (i.e., parallel) than Ecotype × Region interaction effect (i.e., non-parallel) while points falling below the dashed line have stronger Ecotype × Region interaction effect than the Ecotype effect. Correspondingly, Ecotype is non-significant for multiple traits (marked in gray). The traits are categorized according to plant organ to which they belong (different symbols). See Supplementary Table S4 for explanation of individual codes of the traits.

Fourteen of the 16 scored characters significantly differentiated between the ecotypes, however, the level of parallelism remarkably differed across traits (Figure 3). Stem height and traits reflecting flower size consistently varied between ecotypes across regions as demonstrated by significant effect of ecotype but non-significant ecotype × region interaction (GLM, Figure 3, Supplementary Table S9, and Supplementary Figure S5) and strong contribution to the ordination constrained by ecotype (highest loadings to the discriminant axis in LDA, Supplementary Table S10). Overall, alpine plants, regardless of their region of origin, were shorter with larger calyces and petals (Supplementary Table S4). In contrast, traits describing leaf size and shape were those with the strongest ecotype × region interaction indicating region-specific (i.e., non-parallel) morphological differentiation between ecotypes (Figure 3). LDAs run separately for each region revealed such non-parallel response reflected distinct foothill-alpine differentiation in the FG region which was, apart from the height, most strongly driven by traits on leaves (LDA, Supplementary Table S10). Such regionally specific effect was primarily driven by the foothill FG morphotypes which is apparent from their very distinct position in the ordination space (Figure 2C) and higher distinctness of the foothill-FG than the alpine-FG populations when contrasted to populations from other regions belonging to the same ecotype (classification success as an FG group was 86% vs 77% for the foothill vs. alpine populations, respectively).

### Phenotypic Variation in Common Garden

Overall phenotypic differentiation between plants originating from alpine and foothill environment remained highly significant even under two generations of cultivation in a common garden (a subset of 16 populations from four regions; Table 1, Figures 2E,F). Differences between originally foothill and alpine populations were also significant within each region studied (permanova, Table 1), however, with markedly varying strength among the four regions examined, being weakest for NT plants (63% classification success) and strongest for plants from FG region (95%, Table 1). This implies that while alpine populations from some regions (FG and ZT in particular) keep their high morphological distinctness regardless of growth conditions, foothill and alpine populations from the NT region became more similar to each other when grown in a common garden. Morphological disparity of the originally foothill and alpine individuals was still significantly higher than that of their alpine counterparts (*F*_1_,_221_ = 30.96, *p* < 0.001).

Similarly to the field data, traits describing plant height and floral size contributed most strongly to the ecotypic differentiation across all regions (strong effect of ecotype but no ecotype × region interaction; Figure 3 and Supplementary Table S9). On the other hand, ecotypic differentiation disappeared under common garden cultivation for nearly all leaf traits, with the exception of leaf lobe characters. However, leaf lobe traits also exhibited significant region × ecotype interaction due to their strong discriminative power between ecotypes in the FG region but not elsewhere (LDA, Supplementary Table S10).

In summary, reduced stem height and larger floral organs showed the strongest parallelism in the genetic component of morphological differentiation across regions (significant effect of ecotype but non-significant ecotype × region interaction both in field and in the common garden; Figure 3). On the other hand, leaf traits generally showed utmost regionally specific discriminative power in the field (no effect of ecotype and/or non-significant ecotype × region interaction) and such regionally specific discriminative effect mostly disappeared in a common garden, demonstrating plasticity in alpine-foothill differentiation for the majority of leaf traits. The only exception were leaf lobe traits that strongly discriminated foothill and alpine ecotype in one particular region (FG) constantly under both field and common garden conditions, demonstrating genetically determined non-parallelism.

## Discussion

Here, we combined a survey of genetic, ecological and morphological variation in natural populations with a common garden experiment to demonstrate multi-parallel ecotypic differentiation in a wild *Arabidopsis*. Replicated emergence of similar genetically based traits associated with alpine colonization suggests selection triggered by the challenging alpine environment shaped morphological variation of the ancestrally foothill *Arabidopsis* species.

### Parallel Origin of the Alpine Ecotype

A mosaic distribution of ecotypes within a species’ phylogeny, with genetic diversity rather structured by geographical proximity than by environment (the ecotypes), serves as evidence of repeated ecological divergence (Nosil et al., 2008; Johannesson et al., 2010). The clustering, distance-based and coalescent analyses of genome-wide SNPs congruently demonstrated that the sampled *Arabidopsis arenosa* populations exhibit distinct regional clustering regardless of their alpine vs foothill origin in four regions, i.e., supporting the parallel origin scenario. In line with biogeography (Pawłowski, 1970; Mráz and Ronikier, 2016), the major genetic groups corresponded to the four spatially well-defined and floristically distinct mountain regions: Eastern Alps (NT group) and Southern (FG), Eastern (RD) and Western Carpathians (VT + ZT groups). Genetic similarity of Western Carpathian diploids (VT) and tetraploids (ZT) has been detected previously and probably reflects recent origin of the tetraploid cytotype in the area and/or subsequent interploidy gene flow (Arnold et al., 2015; Monnahan et al., 2019; Wos et al., 2019). However, due to distinct ploidy and spatial arrangement reducing the chance for extant gene flow among the VT-alpine and ZT-alpine populations, we considered the two regions as separate units in the following discussion for the sake of clarity.

Parallel origin of the alpine *A. arenosa* ecotype is consistent with previous range-wide studies of the species’ genetic structure (Arnold et al., 2015; Monnahan et al., 2019). Taking into account spatial arrangement of populations and overall phylogeny of the species, colonization of alpine stands from foothill populations likely underlies the observed foothill-alpine differentiation. Firstly, the foothill morphotype represents an ancestral state for the entire species; all three early diverged diploid lineages of *A. arenosa* comprise only foothill populations (Kolář et al., 2016a). Secondly, foothill populations are spread over the entire area of Central and Eastern Europe wherever suitable rocky habitats are available (Kolář et al., 2016b) while the alpine populations are generally rare and confined to isolated spots within the five mountain regions investigated. Presence of such morphologically distinct populations in other areas is highly unlikely given the floristic knowledge of European mountains (Měsíček and Goliašová, 2002; Fischer, 2008; Bartok et al., 2016).

In summary, spatial distribution of genetic variation demonstrated parallel colonization of the alpine habitats by prevalently foothill *Arabidopsis* species in four biogeographically distinct mountain ranges of Central and Eastern Europe.

### Parallel Ecotypic Differentiation

Independent colonization of alpine stands followed by environmentally driven selection should lead to similar phenotypic changes across the alpine populations. Alternatively, drift, divergent selection to locally specific conditions and/or limited variation in the source populations would lead to regionally specific patterns of foothill-alpine differentiation (Bolnick et al., 2018). Independently of the region of origin, alpine *A. arenosa* populations exhibited consistently shorter stems and larger flowers demonstrating parallelism in typical traits associated with “alpine syndrome” that are considered adaptive in alpine environments (Galen, 1989; Halbritter et al., 2018). Elevation gradients belong to the most frequently studied environmental gradients in plant evolutionary ecology since the rise of this field (Turesson, 1930; Clausen et al., 1940), however, cases of phenotypic parallelism demonstrated by a combination of genetic and experimental data are still very rare in plant literature (Fustier et al., 2017). Interestingly, elevational differentiation has been revealed also in other *Arabidopsis* species (Fischer et al., 2013; Günther et al., 2016), often replicated across the studied populations (Kubota et al., 2015; Šrámková-Fuxová et al., 2017; Hämälä et al., 2018), making this genus also an attractive model for the study of alpine adaptation. However, except for European *A. halleri* (Šrámková-Fuxová et al., 2017) we lack a systematic quantitative assessment of morphological variation, leaving mostly unclear to which extent has parallel colonization been accompanied by parallel phenotypic shifts in other *Arabidopsis* species.

Alternatively, morphological similarities among the alpine populations may also reflect plastic response toward similar environmental conditions. To separate both effects, we grew plants of alpine and foothill origin from four regions in a common garden. Originally alpine plants kept their overall distinct appearance even in standard conditions, thus corresponding to the original definition of an ecotype *sensu* Turesson (1922). Importantly, the traits exhibiting the strongest parallelism in foothill-alpine divergence in the field were also highly distinctive in the common garden (stem height and flower size), supporting the hypothesis of parallel selection over phenotypic plasticity. It shall be noted, however, that alternative genetic yet non-adaptive mechanisms such as developmental constraints or mere chance may still stand behind such parallelism (Losos, 2011; Bolnick et al., 2018). In our case, however, we consider the adaptive scenario most likely given the power of four independent transitions ruling out pure chance, very similar direction of the environmental differentiation across regions, and correspondence of our traits with typical “alpine syndrome” observed across floras worldwide (Körner, 2003; Halbritter et al., 2018). In contrast, the regionally specific ecotypic differentiation detected in the field samples disappeared for the majority of such traits in cultivation, suggesting they rather manifested a plastic response to regionally specific conditions. Such plastic response may still represent a way how to cope with the local nuances of the challenging alpine environments (e.g., Anderson and Gezon, 2015), such a hypothesis would, however, require follow-up experimental tests.

### The Degree and Sources of Non-parallelism

Even in systems exhibiting generally strong and genetically determined parallelism, both neutral and selective processes may cause significant non-parallel deviations in particular traits or populations (Stuart et al., 2017; Thompson et al., 2019). Although we observed significant non-parallelism within our set of traits, alpine *A. arenosa* populations showed overall phenotypic homogeneity that was relatively higher than that of their foothill ancestors (as indicated by relatively lower disparity of the alpine ecotype). This contrasts to remarkably divergent phenotypic outcomes of independent shifts along the gradient of elevation in the other multi-parallel plant system, *Heliosperma* (Bertel et al., 2018).

For most of the traits exhibiting non-parallelism in the field data, ecotypic differentiation was no longer present under cultivation in the common garden, demonstrating phenotypic plasticity is the likely major driver of non-parallelism in our data. Only in one case (FG region) an additional trait (leaf lobes) discriminated foothill and alpine populations both in the field and in the common garden suggesting genetic determination of this component of non-parallelism. We consider a neutral scenario that initially divergent variation of the source (foothill) populations is likely responsible for this difference. Notably, the FG-foothill populations were more strongly phenotypically divergent from the other foothill regions than were their FG-alpine counterparts from the other alpine populations. Emergence of non-parallel traits in response to local adaptation is less likely due to overall lower phenotypic variation among the alpine populations and very similar environmental conditions across alpine sites, speaking against strong selection triggered by locally specific conditions. As parallelism informs about the role of selection, its detection is usually the prime aim of most studies (Sackton and Clark, 2019), although the regionally specific deviations provide valuable information on additional evolutionary processes affecting the predictability of evolution (Stuart et al., 2017).

## Conclusion

Our study demonstrated that spatially isolated alpine environments harbor populations exhibiting remarkable phenotypic parallelism that is manifested by traits that are associated with alpine adaptation in general. Replicated phenotypic shifts in response to similar environmental pressures that are stable over two generations in cultivation suggest the role of selection triggered by the stressful alpine environment. Our findings open new avenues for studying convergent genetic underpinnings of phenotypic parallelism currently being addressed by a follow-up study (Bohutínská et al., 2020). Indeed, alpine *A. arenosa* is a particularly well-suited model for following such questions due to negligible neutral divergence between ecotypes within each region and lack of strong bottleneck, both of which mitigate potentially confounding signals of past demographic events. This, complemented by the genomic and genetic tractability of the *Arabidopsis* genus in general and this species in particular (highly variable outcrosser) make *Arabidopsis arenosa* a highly promising model for studying the genomic basis of parallel adaptation in plants.

## Data Availability Statement

The datasets presented in this study are provided as Supplementary Datasets (environmental parameters and morphological traits) and in online repositories (DNA sequences): NCBI BioProjects, accession nos: PRJNA484107 and PRJNA633005.

## Author Contributions

FK and KM designed the study. AK, MB, GŠ, DP, FK, and KM collected the data. AK, VK, GW, and VZ analyzed the data. FK and AK drafted the manuscript with contribution of all authors. All authors contributed to the article and approved the submitted version.

## Conflict of Interest

The authors declare that the research was conducted in the absence of any commercial or financial relationships that could be construed as a potential conflict of interest.
