# Supplementary material for: Parallel Alpine Differentiation in Arabidopsis arenosa
Source: Front Plant Sci. 2020 Dec 8;11:561526. doi: 10.3389/fpls.2020.561526 (PMC7753741; doi:10.3389/fpls.2020.561526)
Supplement: Supplementary file 1 [file Data_Sheet_1.pdf]

Parallel alpine differentiation in *Arabidopsis arenosa*

Adam Knotek, Veronika Konečná, Guillaume Wos, Doubravka Požárová, Gabriela Šrámková, Magdalena Bohutínská, Vojtěch Zeisek, Karol Marhold, Filip Kolář

Methods S1*: Experimental cultivation conditions*

We firstly raised one generation of plants from each population in growth chambers under constant conditions. The field-collected seeds were germinated on a mixture of peat free potting compost and white river sand fraction 16-22 mm of neutral reaction in 2/3 ratio. Seedlings were re-potted at 2-4 leaves stage to 750 cm^3 pots with the same substrate, young rosettes were vernalized for 11 weeks (4 °C, 8/19 hrs day/night cycle) and then transferred to standard conditions in the chamber (21/18 °C, 16/8 hrs day/night cycle, light ~300 umol m-2*s-1). Each plant was hand-pollinated by a mixture of pollen from the same population (~ 14 flowering plants per population were used) for a period of one week. Ripe seeds (representing a genetically variable mixture of full- and half-sibs) were harvested and stored at dark and dry place at 4 °C.

The plants used for phenotyping were raised in the Botanical Garden of the University of Innsbruck, Austria (600 m a. s. l.; 47.26773 N, 11.37898 E). Seeds from min 10 maternal plants per population were sown in May 2018 on filter papers imbibed with 5 mM potassium nitrate and submitted to 4 days of cold stratification (4°C) before transfer to a growth chamber at 25 °C under constant light (~300 µmol $m^{-2}*s^{-1}$). Upon germination, seedlings were transferred to multi-pot trays using a mixture of peatless garden substrate and sieved sand of neutral reaction (1/1) and kept in an open-air greenhouse. Seedlings were then transplanted into individual pots (7 cm diameter, about 160 cm^3) filled with a mixture of rough-texture soil (5 parts of leaf compost, 2 parts of ground soil, 1 part of horticultural lava, 2 parts of coconut fibre, 2 parts of sand and 0.2 parts of stone powder) of neutral reaction (8/10), vermiculite (1/10) and sieved sand (1/10). All plants were kept in a sheltered outdoor place in the botanical garden over winter and the rosettes with set-up buds were brought back to the open-air greenhouse in mid-March 2019. Plants were watered regularly throughout the experiment. Finally, after four weeks the phenotypic traits were collected on all plants in the full-flowering stage.

Methods S2: *Sequencing, raw data processing, variant calling and filtration*

RADseq data

One methylation-insensitive restriction enzyme (HpyCH4V) was used to generate blunt-end DNA fragments, followed by an A-tailing step combined with ligation of constructed adapters with T overhang and custom barcodes. Libraries were sequenced on an Illumina Hiseq 2500 platform using 125-bp paired-end reads at EMBL Genomics Core Facility (Heidelberg, Germany). Raw reads were demultiplexed, quality trimmed (> 30 Phred quality score) and mapped using BWA v. 0.7.3a on *Arabidopsis lyrata* reference v. 1.0.25 and the alignment was further processed with Picard Tools v. 1.119. The Genome Analysis Tool Kit v. 3.8-1 was used for simultaneous discovery and genotyping of SNPs and for calling of invariant sites following the recommended best practice (www.broadinstitute.org/gatk/). GATK performs SNP discovery and probabilistic genotype calling across all samples simultaneously, which is a more accurate than individual-based SNP calling. Namely, we used HaplotypeCaller module to call variants per individual with respect to its ploidy level (i.e., using the ploidy = 4 option which enables calling full tetraploid genotypes). Then we aggregated variants across all individuals by module GenotypeGVCFs. We retrieved SNP data for sites corresponding to the called RAD loci from a set of genome resequencing data of an additional 11 populations (mapped to the same reference and scored in the same way as described above, sequences available at Sequence Read Archive, SRA, code PRJNA484107). To do so, we extracted coordinates of the RAD loci, i.e. variant and invariant sites that were called in at least 30 % of individuals. Using these coordinates, we extracted the same sites from the filtered genomic vcf and combined both vcfs into a single file (CombineVariants). This merged raw vcf file containing the final set of 200 individuals from 57 populations was further filtered as follows: using GATK, we retained only biallelic sites that mapped to nuclear chromosome scaffolds with a minimum mapping quality of 40, which did not show mapping quality bias for the reads supporting the non-reference allele (keeping only variants with mapping quality rank sum test value higher than -12.5) and which were present in at least 80 % of our individuals at a sequencing depth of 8× or greater. In addition, we excluded

potentially paralogous sites by masking genes that showed excess heterozygosity in a set of genome-resequenced populations from throughout the range of *A. arenosa*. We also masked sites that had excess read depth that we defined as 1.6× the second mode of the read depth distribution in the same genomic dataset.

Methods S3: *Coalescent simulations*
We inferred the likely scenario of the origin of alpine populations using coalescent simulations performed in *fastsimcoal v.26* (version for linux 64-bit) run at Metacentrum computing cluster (https://metavo.metacentrum.cz/). We aimed to discriminate between single versus parallel origins of alpine ecotype, optionally assuming gene flow between ecotypes within each region, using population quartets involving one alpine and one foothill population from two regions. This strategy gave us four scenarios to test (single origin vs. parallel origin each with or without bi-directional between-ecotype admixture, see Fig. 1C), each of which was compared with observed data gathered for all six possible pairs of regions occupied by tetraploid populations, i.e. six quartets of populations from NT-RD, NT-FG, NT-ZT, RD-FG, RD-ZT, FG-ZT regions (i.e., 24 sets of simulations in total) and a pair of spatially closest diploids and tetraploids (VT-ZT). In order to keep the number of simulations realistic, the quartets involving diploids (VT) and other tetraploid regions were not simulated, as single origin of the tetraploid cytotype inferred previously makes origin of alpine tetraploids via independent polyploidisation from alpine VT diploids very unlikely.

Each model was fit to a multi-dimensional allele frequency spectrum calculated from putatively neutral four-fold degenerate SNPs. To gather robust allele frequency spectra, we acquired genome-wide SNP variation from a subset of sufficiently sampled populations (genome resequencing data, 7-8 individuals per population, 176,000 – 417,000 SNPs per population; Table S2), one alpine and one foothill per each region. We used populations for which genome resequencing data were available from a previous range-wide study (Monnahan et al. 2019, PRJNA484107) and complemented them with newly sequenced data (SRA project SUB6592572; see Table S2 for details). We extracted the four-fold degenerated SNPs (inferred based on *A. lyrata* annotation) using custom python scripts available at https://github.com/mbohutinska/ScanTools_ProtEvol, the allele frequency spectra, stored in *.*dsfs* files, are attached as Supplementary data file S1. Note that identical SFS were used for all scenaria within particular population quartet, the used *.*dsfs* files differ only by their name, adhering to a naming convention for purposes of batch analysis by the script. Read trimming, mapping, variant calling and filtration was performed as described above for RADseq data. We polarized the sites using a collection of genotyped individuals across closely related diploid *Arabidopsis* species, thus avoiding polarization against a single individual (the reference *A. lyrata* individual) following Monnahan et al. (2019).

For each scenario and population quartet, we performed 50 independent fastsimcoal runs to overcome local maxima in the likelihood surface. We run fastsimcoal analysis in batches using the python script FSC2_submit.py from ScanTools_ProtEvol toolset, with one generic *.*est* and *.*tpl* file for each model, as the submission script automatically assigns sample sizes based on the inputted allele frequency spectra (the script, readme file, generic *.*est* and *.*tpl* files as well as all *.*dsfs* input files and **par* files representing outputs of the run with highest likelihood for each of the 28 combinations are available in the Supplementary data file S1). We used the following options for each fastsimcoal run: *fsc26 -t Name_of_scenario.tpl -e Name_of_scenario.est -n 100000 -u -q -L 40 -M*. We performed (-n) 100000 simulations to estimate the expected SFS in each cycle and run (-L) 40 optimization (ECM) cycles to estimate the parameters. For divergence times, we set the upper limit (used only as a starting value for searching in fastsimcoal) of the ancestral split to 30k generations representing the upper limit of estimated time of the single origin of the tetraploid cytotype inferred from genome-wide data (i.e. conservative value, the range 20-31 k generations inferred over multiple population quartets using range-wide sampling by Monnahan et al. (2019). Consequently, we set the upper limit of the divergence time between the sister populations to 10 k generations, corresponding to LGM (assuming generation time of 2 years) as all sampled alpine populations occupy mountain tops and glacial cirques, i.e. habitats that have been glaciated during LGM. We assumed mutation rate of 4.3e-8 inferred for *A. arenosa* previously (Arnold et al., 2015). We then extracted the best likelihood partition for each fastsimcoal run, calculated Akaike information criterion (AIC, following

Arnold et al., 2016) and summarized them across the 50 different runs. The scenario with consistently lowest AIC values within particular population quartet was preferred (Fig. S3).

References

Arnold B. J. et al. Borrowed alleles and convergence in serpentine adaptation. *Proc. Natl Acad. Sci. USA* 113, 8320–8325 (2016).

Monnahan P. et al. Pervasive population genomic consequences of genome duplication in *Arabidopsis arenosa*. Nature Ecology & Evolution. 3:457 (2019).

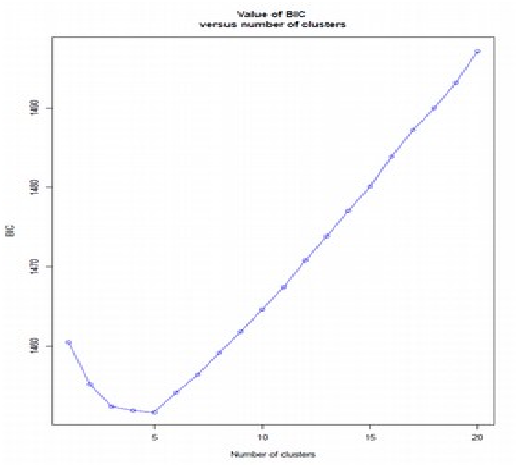

Fig. S1 Selection of plausible number of clusters (K) in K-means clustering of SNP data. Bayesian information criterion (BIC) of K-means groupings from K=1 to K=20 summarized over 1000 random starts.

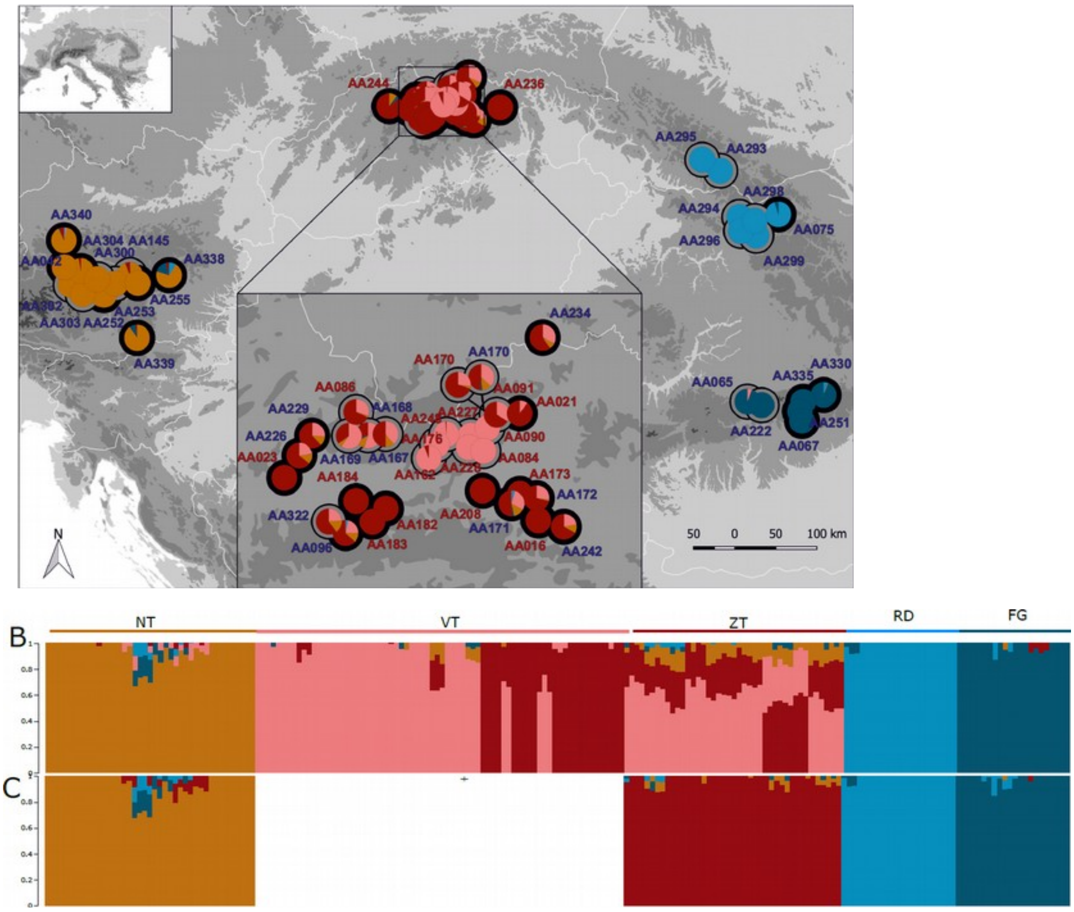

Fig. S2 Genetic structure of alpine and foothill *Arabidopsis arenosa* identified by FastStructure. (A) Geographic distribution of the five genetic groups identified by FastStructure (different colours) and the two altitudinal ecotypes (different shading of circle margins: black = foothill, gray = alpine). Pie charts reflect assignment to different FastStructure groups, population labels are coloured by ploidy level (red = diploid, blue = tetraploid). The inset depicts detailed distribution in the Western Carpathians (a diploid VT and tetraploid ZT groups) (B) Posterior probability of assignment of all sampled *A. arenosa* individuals to the five groups as inferred by FastStructure under K=5. (C) Posterior probability of assignment of the tetraploid *A. arenosa* individuals to the four groups (four tetraploid regions only) as inferred by FastStructure under K=4.

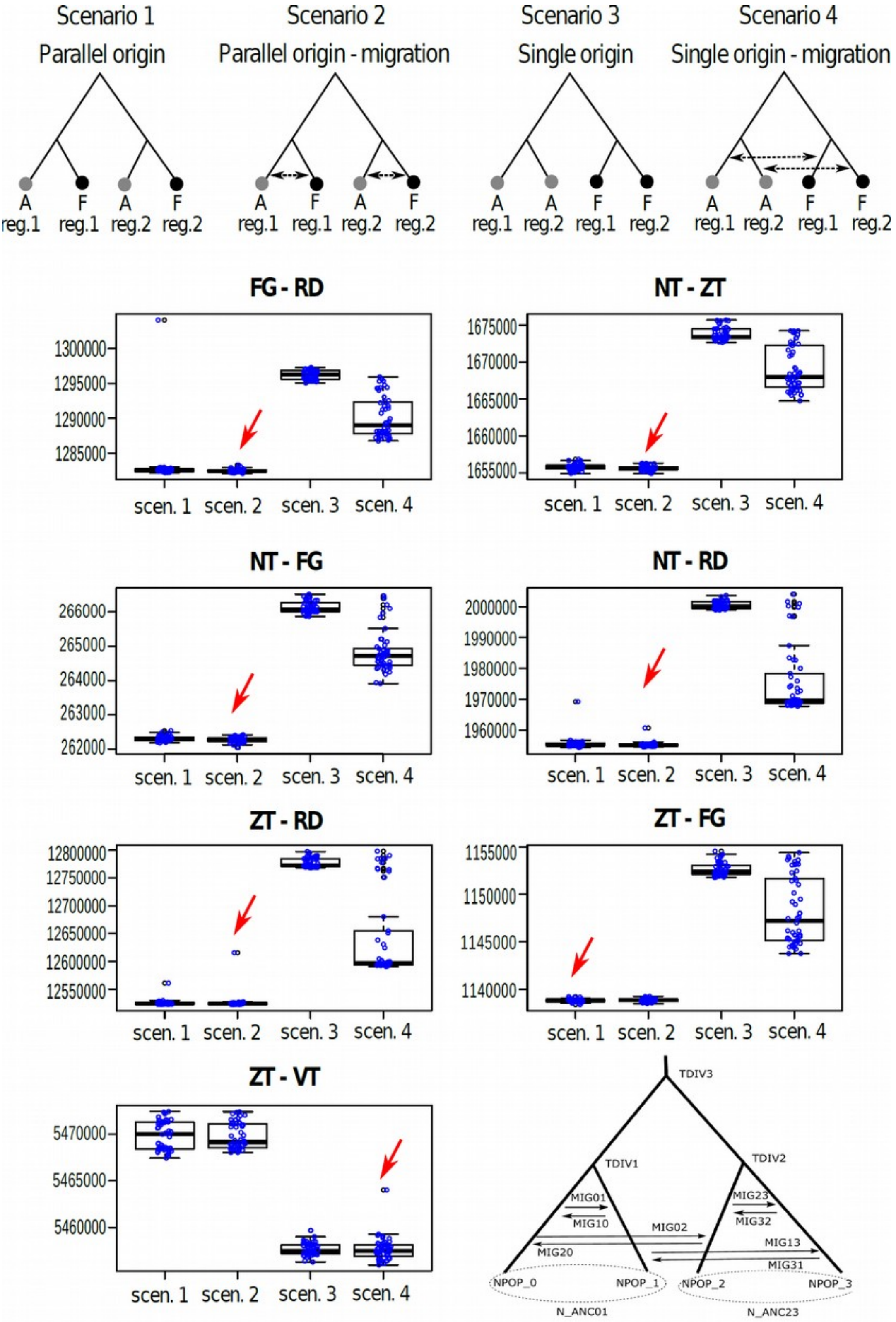

Fig. S3 Comparison of Akaike information criteria (AIC) across four scenarios approximating the origin of the alpine ecotype of *A. arenosa* in pairs of mountain regions. We iterated alpine (A) and foothill (F) pops. from the following regions (reg.): FG, NT, RD, VT and ZT. Each scenario was simulated by 50 fastsimcoal2 independent runs, the corresponding distribution of the AIC values over these 50 runs (blue dots) is summarized by the boxplots. Red arrow highlights the most likely scenario with the lowest median AIC values. The topologies of the evaluated scenarios are depicted above the corresponding plots, model parameters are described in the bottom right scheme.

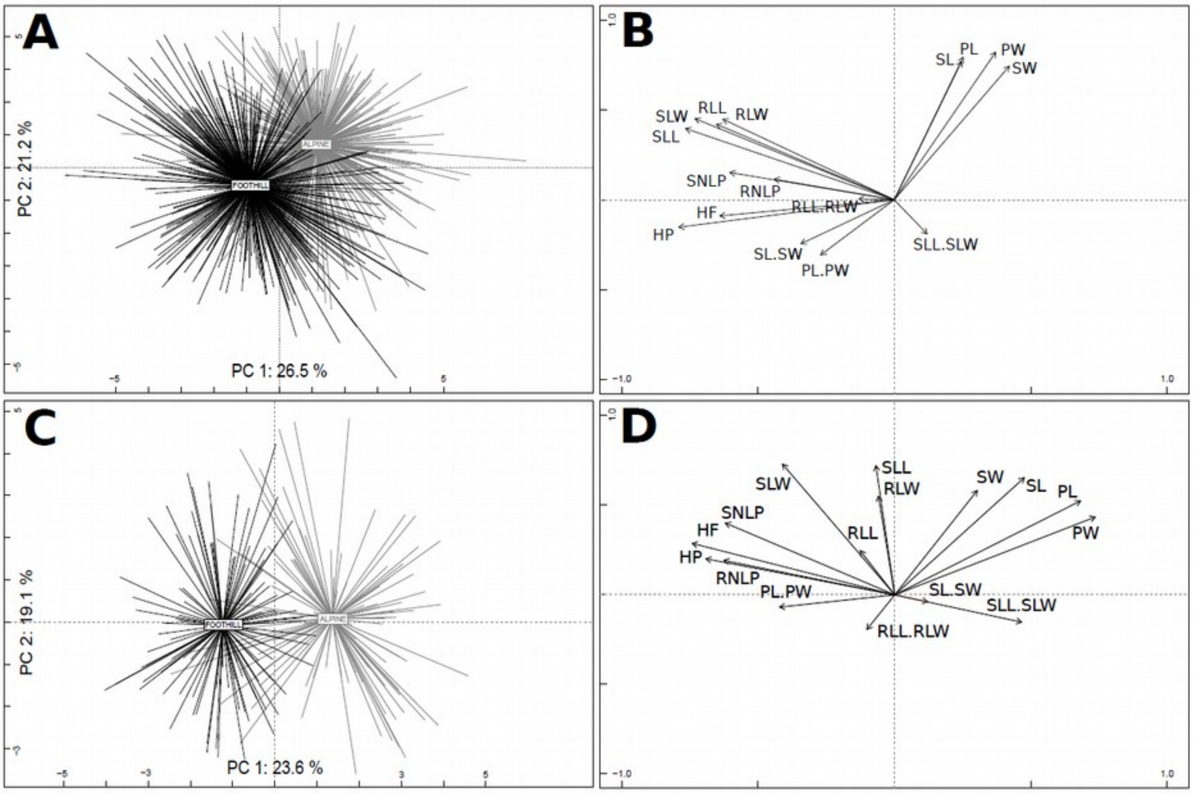

Fig. S4 Morphological differentiation among foothill (black) and alpine (gray) individuals. Principal component analysis was run using 16 morphological traits based on (A, B) 999 individuals collected in field in five regions; (C, D) 223 individuals from the common garden experiment originating from four regions. Contribution of morphological characters for (B) plants collected in the field (D) plants from common garden experiment.

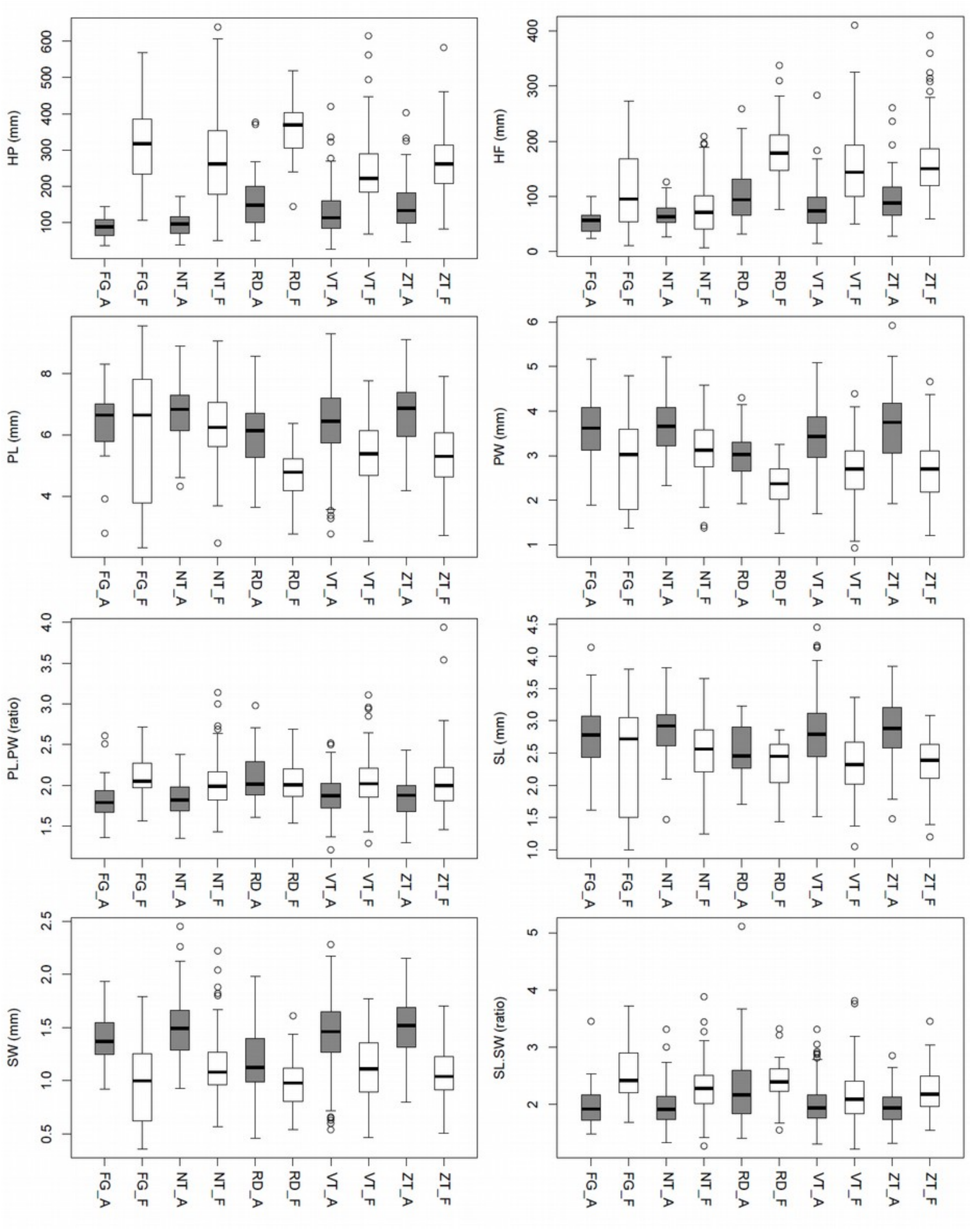

HP (mm)
HF (mm)
PL (mm)
PW (mm)
PL.PW (ratio)
SL (mm)
SW (mm)
SL.SW (ratio)
FG_A FG_F NT_A NT_F RD_A RD_F VT_A VT_F ZT_A ZT_F

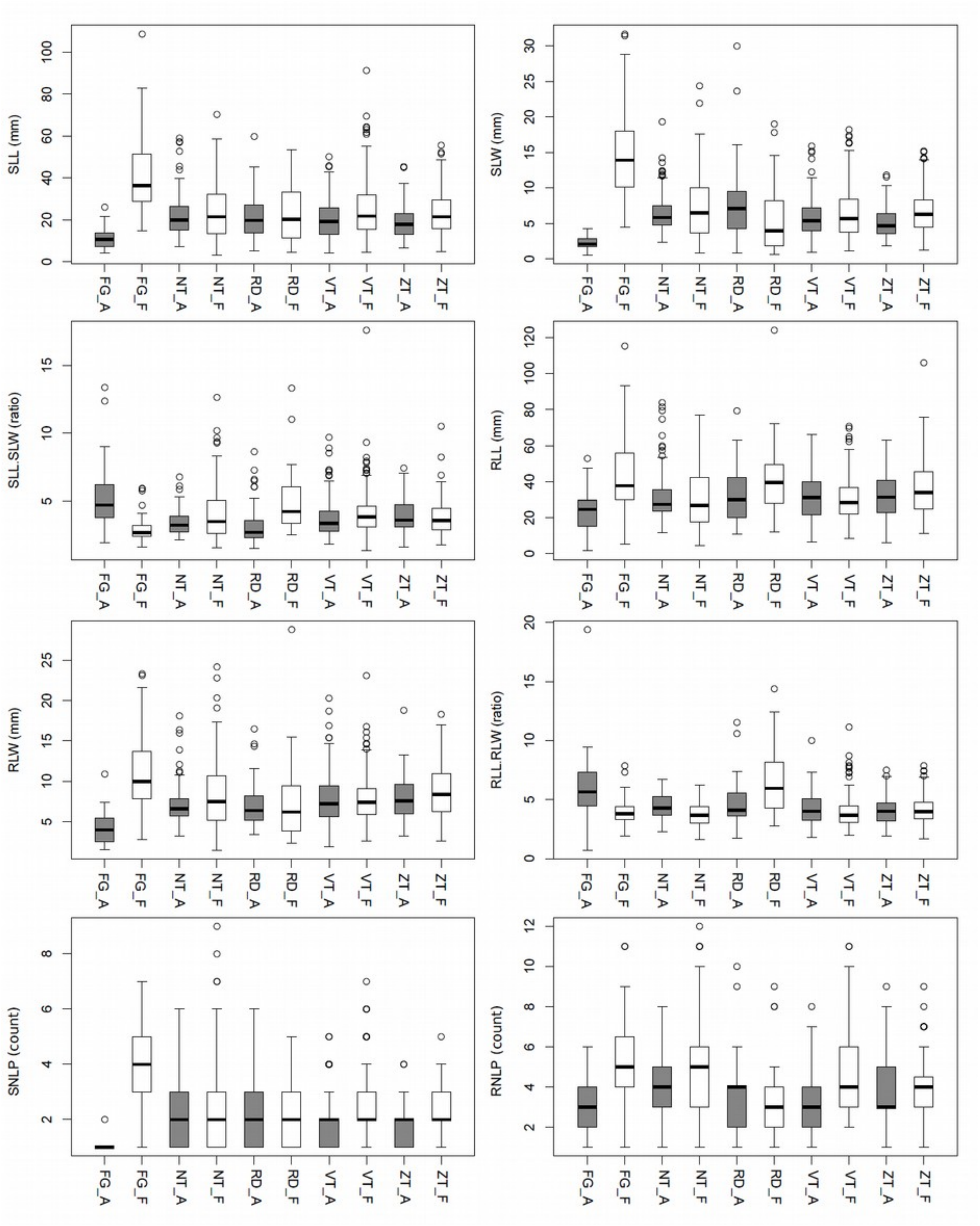

Fig. S5 Distribution of trait values in foothill (F, white) and alpine (A, gray) groups of *A. arenosa* plants sampled in field in the five regions (FG, NT, RD, VT, ZT). Original values are provided in a Supplementary data file S3.

Table S1 Details on ploidy, sampling location, habitat and genetic diversity of all populations sampled for the study.

Population code	Ecotype	Region	Ploidy	Kmeans_group	N_morphology (field)	N genetic	Common Garden experiment	State	Locality	Collected	Latitude	Longitude	Elevation	Genetic diversity	Soil pH
AA016	F	VT	2x	4	20	8	15	SK	Košický kraj: Podlesok, rocks in the entrance to Suchá Belá gorge, limestone rocks	E. Záveská, J. Kučera, F. Kolář	48.9603	20.3833	600	0.051	7.82
AA021	F	VT	2x	4	20	3		SK	Prešovský kraj: Tatranská kotlina, rocks next to the entrance to Belianska jaskyňa cave, slopes and limestone rocks in mixed forest	E. Záveská, F. Kolář	49.2289	20.3117	900		7.26
AA023	F	VT	2x	4	19	2		SK	Žilinský kraj: Bešeňová, travertine rock 1 km NNE from the church in the village, travertine rock	E. Záveská, F. Kolář	49.1073	19.4347	574		7.28
AA042	F	NT	4x	2	20	3		AT	Steiermark: Öblarn, slopes of valley of Walchenbach S of the village, rocky valley	F. Kolář	47.4508	14.0064	713		7.52
AA065	A	FG	4x	3	15	4 (BAL)	13	RO	Argeş: Făgăraş Mts, slopes above lake Balea, close to Transfăgăraş road, alpine scree	F. Kolář, G. Šrámková	45.602	24.6226	2269		5.58
AA067	F	FG	4x	3	20	4 (DRA)	13	RO	Argeş: Dâmbovicioara, river canyon W of the village, shady rocks in a canyon	F. Kolář, G. Šrámková	45.4416	25.2239	858	0.046	7.65
AA075	F	RD	4x	1	15	4		RO	Suceava: Cârlibaba, rocky slope above the road to Borşa, W of the village, shady rocks, eroded slope	F. Kolář, G. Šrámková	47.5759	25.0771	981	0.058	7.56
AA084	A	VT	2x	5	20	4 (VEL)	8	SK	Prešovský kraj: Vysoké Tatry, wet rocks and gravel in Velická Dolina ravine, between Sliezsky dom chalet and Dlhé pleso lake, alluvial gravel, wet rocks	F. Kolář, E. Záveská, S. Španiel, M. Kolník, K. Marhold	49.162	20.1542	1823	0.048	5.23
AA086	A	VT	2x	4	12	2		SK	Žilinský kraj: Zuberec, Zverovka, limestone rocks at the top of Osobitá mountain, exposed rocks, gravel	F. Kolář, E. Záveská, S. Španiel, M. Kolník, K. Marhold	49.2593	19.7218	1552		6.11
AA090	A	VT	2x	5	17	4	11	SK	Prešovský kraj: Vysoké Tatry, screes and rocks above the Zelené pleso lake up to Medený vodopád waterfall and Dlhý vodopád waterfall, scree, wet rocks	F. Kolář, E. Záveská, S. Španiel, M. Kolník, K. Marhold	49.2065	20.2151	1625	0.048	5.53

AA091	A	VT	2x	4	20	3		SK	Prešovský kraj: Ždiar, Belianské Tatry, along blue tourist path in Kopské sedlo pass, exposed rocks, eroded slope	F. Kolář, E. Záveská, S. Španiel, M. Kolník, K. Marhold	49.2299	20.219	1751		7.14
AA096	F	ZT	4x	4	19	4		SK	Žilinský kraj: Nízke Tatry, Liptovský Ján, Jánska dolina valley, S of the village, alluvial gravel, eroded slope above road	F. Kolář, S. Španiel, M. Kolník, K. Marhold; J. Mořkovská	49.0108	19.6733	744	0.045	7.15
AA145	A	NT	4x	2	19	3		AT	Steiermark: Niedere Tauern, valley and rocks along the trekking path towards Gamskögel mountain, moist valley (with Petasites sp.) and rocks	E. Záveská, S. Španiel	47.3733	14.554	1752		NA
AA162	A	VT	2x	5	20	2		SK	Prešovský kraj: Vysoké Tatry, Mlynická dolina valley, Skok waterfall, wet rocks, alluvial gravel	F. Kolář, M. Lučanová, K. Marhold, J. Smatanová, J. Mořkovská	49.1534	20.0458	1750		5.66
AA167	A	ZT	4x	4	16	3		SK	Žilinský kraj: Pribylina, Račkova dolina valley and Račkove plesá lakes, open places in grasslands disturbed by marmots	M. Lučanová, K. Marhold	49.2	19.8047	1690		5.16
AA168	A	ZT	4x	5	12	4 (TKO)	14	SK	Žilinský kraj: Zuberec, Zverovka, rocky slopes above Horné Roháčske pleso lake and on N slopes of Tri Kopy mountain, wet rocks, screes	F. Kolář, K. Marhold	49.2045	19.7352	1783	0.052	4.83
AA169	A	ZT	4x	5	12	3	15	SK	Žilinský kraj: Vysoké Tatry, northern rocky slopes under the Baníkovské sedlo saddle, wet rocks	M. Lučanová, J. Smatanová, J. Mořkovská	49.2012	19.7084	1864		5.31
AA170_2	A	VT	2x	4	28	4 (TRD)		SK	Prešovský kraj: Žiar, Tristárska dolina valley, open gravely sites, screes	F. Kolář, M. Lučanová, K. Marhold, J. Smatanová, J. Mořkovská	49.2502	20.2053	1650	0.051	6.31
AA170_4	A	ZT	4x	4	20	4 (TRT)		SK	Prešovský kraj: Žiar, Tristárska dolina valley, open gravely sites, screes	F. Kolář, M. Lučanová, K. Marhold, J. Smatanová, J. Mořkovská	49.2502	20.2053	1809	0.05	7.51
AA171	F	ZT	4x	4	19	4	15	SK	Prešovský kraj: Hranovnica, slopes above the road to Poprad town, S of the Kvetnica village, rocky eroded slope above the road and open oak forest	F. Kolář, M. Lučanová, J. Mořkovská	49.0072	20.2864	720	0.057	6.46
AA172	F	ZT	4x	4	20	3		SK	Prešovský kraj: Primovce, Primovské skaly rocks in the village, shady rocks	F. Kolář, M. Lučanová, J. Mořkovská	49.0159	20.3825	605		5.39
AA173	F	VT	2x	4	17	3		SK	Prešovský kraj: Gánovce, travertine outcrop in the village, exposed rocks	F. Kolář, M. Lučanová, J. Mořkovská	49.0299	20.3207	664		7.29
AA176	A	VT	2x	5	17	3		SK	Prešovský kraj: Vysoké Tatry, Mengusovská dolina valley, Veľké Hincovo pleso lake, open moist gravely patches, lake shore	F. Kolář	49.1756	20.0605	1950		6.01

AA182	F	VT	2x	4	20	3		SK	Žilinský kraj: Kráľova Lehota, rock above Čierny Váh river, exposed dry rock	K. Marhold, J. Smatanová; J. Mořkovská	49.0153	19.8111	670		6.94
AA183	F	VT	2x	4	20	4		SK	Žilinský kraj: Malužiná, slope above road to the quarry, eroded slope	K. Marhold, J. Smatanová; J. Mořkovská	48.9845	19.7573	735	0.045	7.24
AA184	F	VT	2x	4	19	3		SK	Žilinský kraj: Liptovský Hrádok, slopes above Čierny Váh river, near Borová Sihoť spring, eroded slope	K. Marhold, J. Smatanová; J. Mořkovská	49.0386	19.7005	624		7.21
AA208	F	VT	2x	4	38	8	14	SK	Prešovský kraj: Svit, Baba hill, SE slopes, forest clearing in pine forest	J. Mořkovská, J. Smatanová	49.0435	20.1808	844	0.05	7.54
AA220	F	RD	4x	NA	12	0		RO	Bistrita-Năsăud: Parva, slopes above the road in a deep valley of Rebra brook at N margin of the village, open sites above road in deciduous forest	F. Kolář, M. Bohutínská	47.4024	24.5461	545		6.09
AA222	A	FG	4x	3	16	4 (LAC)	15	RO	Argeş: Făgărăş Mts., slopes above path from second bend of the Transfăgărăşan road S of the tunnel to Lacul Capra lake, calcareous rocks and scree	F. Kolář, M. Bohutínská	45.5954	24.6346	2092	0.042	7.5
AA226	F	ZT	4x	4	12	3		SK	Žilinský kraj: Prosiek, transect through Prosiecka dolina valley, N of the village, semi-shady rocks in a gorge	J. Mořkovská	49.16	19.4962	656		7.19
AA227	A	VT	2x	5	21	2		SK	Prešovský kraj: Vysoké Tatry, ca 300 meters E of Prielom saddle, along the blue marked tourist path, alpine scree	J. Mořkovská	49.1762	20.15	2070		6.94
AA228	A	VT	2x	5	17	3		SK	Prešovský kraj: Vysoké Tatry, north facing slope below Pod Polskym hrebeňom saddle, along tourist path, rocks	J. Mořkovská	49.1738	20.1391	2147		5.19
AA229	F	ZT	4x	4	15	4	15	SK	Žilinský kraj: Huty, rocks in the entrance to Kvačianska dolina gorge, limestone rocks	E. Záveská, J. Kučera, F. Kolář	49.2076	19.5494	750	0.038	7.17
AA234	F	ZT	4x	4	19	4		SK	Prešovský kraj: Lesnica, N facing slopes of the Dunajec river canyon 1.5 km NW of the village, shady rocks in beech forest	F. Kolář, G. Šrámková, J. Smatanová	49.4117	20.4488	437	0.044	7.26
AA236	F	VT	2x	4	19	4		SK	Prešovský kraj: Lipovce, at the lowest waterfall in Kamenná Baba gorge, 1.5 km W of the village, shady limestone rocks	F. Kolář, G. Šrámková, J. Smatanová	49.0589	20.9306	659	0.042	6.82
AA242	F	ZT	4x	4	20	3		SK	Košiský kraj: Spišká Nová Ves - Čingov, N facing slopes above Hornád river, 500 m WSW of the settlement, shady north-facing rock	J. Mořkovská, K. Marhold, S. Španiel, J. Smatanová	48.9402	20.4774	530		7.29
AA244	F	VT	2x	4	12	3		SK	Žilinský kraj: Šútovo, small limestone hill at E margin of the village, rocky and gravely sunny slope	F. Kolář, A. Knotek, G. Šrámková	49.15197	19.085165	480		7.41
AA248	A	VT	2x	5	4	4		SK	Prešovský kraj: Rysy mountain, just below the summit, rocky outcrop near the summit	M. Bohutínská	49.1795	20.0881	2488	0.041	5.43

AA251	F	FG	4x	3	14	3	15	RO	Argeş: Dâmbovicioara, river canyon S of the village, gravel along the road, most likely secondary habitat, not on the rocks only below them in the gravel	E. Záveská	45.4267	25.2133	915		8.3
AA252	F	NT	4x	2	19	3	15	AT	Steiermark: Schönberg-Lachtal, along a road L 514, ca 600 m N of of Hocheggerstraße and Glattjoch Straße junction, gravel and stones along the road	F. Kolář, M. Hanzl, E. Záveská, S. Španiel	47.1826	14.3379	856		8.02
AA253	A	NT	4x	2	21	4 (SCH)	15	AT	Steiermark: Niedere Tauern, Schießeck mountain, N exposed rocky slopes, screes and rocks, quartzite	F. Kolář, M. Hanzl, S. Španiel	47.2777	14.3219	2240	0.033	6.7
AA254	A	NT	4X	NA	0	0	15	AT	Steiermark: Seckauer Alpen: Hochreichart, northwestern crest, stabilized amphibolite screes	P. Schönswetter	47.36444	14.68083	2360		NA
AA255	F	NT	4x	2	20	4	15	AT	Steiermark: Seckauer Alpen, lower-most Ingeringgraben, 1.6 km NNW of Wasserberg castle, Ingeringbach valley, siliceous rocks	P. Schönswetter	47.2842	14.6819	970	0.052	4.72
AA293	A	RD	4x	1	4	3		UA	Zakarpatská oblast: Dragobrat, bellow the top of Blyznytsya mountain, S slope, in glacial cirque, 70 - 100 vertical meters above Ivor lakes, limestone in glacial cirque	M. Bohutínská, J. Hojka, D. Bohutínský, F. Rooks	48.2286	24.2323	1614		5.44
AA294	A	RD	4x	1	4	3		RO	Maramureş: Borşa, around the entrance to glacial cirque at E slopes of Pietrosul Rodnei mountain, along the dirt road, 5.7 km SSW of the town, rocky slope with sparse vegetation	F. Kolář, M. Bohutínská	47.6031	24.6488	1780		6.98
AA295	A	RD	4x	1	12	3		UA	Zakarpatská oblast: Svidovec, Gerisaska mountain, upper part of the glacial cirque (below the rocks) on the eastern slope, rocks and scree in glacial cirque	J. Chrtek, K. Kabátová	48.2722	24.1623	1702		6.38
AA296	A	RD	4x	1	10	3		RO	Maramureş: Borşa, rocks at the NW facing slopes of glacial cirque at E slopes of Pietrosul Rodnei mountain, 6 km SSW of the town, rocky crevices	F. Kolář, M. Bohutínská	47.5977	24.6469	1820		5.15
AA298	A	RD	4x	1	18	4		RO	Maramureş: Borşa, NW slopes of Vârful Ciungilor mountain, in Bila stream and on the rock above spring of the stream, limestone	M. Bohutínská, J. Hojka, D. Bohutínský, F. Rooks	47.5393	24.8868	1586	0.06	6.63
AA299	A	RD	4x	1	10	2		RO	Maramureş: Borşa, NW slope of the NW glacial cirque bellow Ineu peak, glacial cirque	M. Bohutínská, J. Hojka, D. Bohutínský, F. Rooks	47.5273	24.8806	2017		7.12
AA300	A	NT	4x	2	18	4		AT	Steiermark: Pusterwald, shores of Wildsee lake below Eiskarspitz mountain, snowbed in a glacial cirque	F. Kolář, A. Knotek, S. Španiel, P. Schönswetter, K. Hülber	47.3256	14.2304	2117	0.056	6.02
AA302	A	NT	4x	2	17	3		AT	Steiermark: Krakaudorf, screes in Sauoffensee glacial cirque, W of the lake, wet rocks and scree slope below	F. Kolář, P. Schönswetter, K. Hülber	47.2586	14.0103	2184		6.16

AA303	A	NT	4x	2	20	2		AT	Steiermark: Krakaudorf, slope above Sauoffensee lake, stony snowbed slope	A. Knotek, S. Španiel	47.2588	14.0049	2030		5.54
AA304	F	NT	4x	2	20	3		AT	Steiermark: Aigen, rocks in Gullingtal valley, next to the bridge W of a quarry, rocks	F. Kolář, A. Knotek, S. Španiel	47.4936	14.1722	800		7.74
AA322	A	ZT	4x	4	20	3		SK	Nizke Tatry: NE slope between Krakova hola mountain and Pusté mountain, rocky sites in forest clearing	J. Mořkovská	48.9919	19.623	1472		7.07
AA330	F	FG	4x	3	13	4		RO	Brasov: Timisu de Sus, rocks above railway, ca 5 km NNE of the village, shady rocks, travertine spring	F. Kolář, M. Bohutínská, D. Požárová, F. Rooks	45.57	25.6083	797	0.063	8
AA335	F	FG	4x	3	20	3		RO	Brasov: Zărneşti, narrowest part of Prăpăstiile Zărneştilor gorge, 5.5 km SW of the railway station in the town, rocks in the gorge	F. Kolář, M. Bohutínská, D. Požárová, F. Rooks	45.5253	25.2812	978		6.76
AA338	F	NT	4x	2	20	4 (HOC)		AT	Steiermark: Mautstatt, Calcareous rocks and screes near road and railroad, ca 4 km NE from the village, calcareous rocks and bank between railroad and small river	A. Knotek; D. Požárová	47.37	15.3867	560	0.044	7.61
AA339	F	NT	4x	2	18	4 (KAS)		AT	Kärnten: Sankt Paul im Lavanttal , castle ruin Rabenstein, circa 2 km S from the town, rocks and walls of castle ruins	A. Knotek; D. Požárová	46.6883	14.8717	620	0.045	7.98
AA340	F	NT	4x	2	20	4 (KOS)		AT	Oberösterreich: Mitterweißenbach, Kesselbach stream riverbank near the road from Bad Ischl to Ebensee, calcareous river bank	A. Knotek; D. Požárová	47.7469	13.6897	460	0.048	8.42

Table S2 Details on diversity and accession codes of genome resequenced populations used for coalescent simulations

pop code	Region	Ecotype	N indivs	N all sites called	N SNPs	Genetic diversity (pi)	original code	BioSample codes (SRA)
AA300	NT	Alpine	8	3939154	361293	0.0267	WIL	SAMN13420543-50
AA339	NT	Foothill	8	2920550	262141	0.0255	KOS	SAMN09759803-09
AA065	FG	Alpine	8	3950174	349273	0.0260	BAL	SAMN13420559-66
AA067	FG	Foothill	8	2218553	176496	0.0213	DRA	SAMN09759740-47
AA299	RD	Alpine	7	3925916	417156	0.0286	INE	SAMN13420575-81
AA075	RD	Foothill	8	3917955	409493	0.0213	CAR	SAMN13420582-89
AA168	ZT	Alpine	8	3723199	354763	0.0285	TKO	SAMN09759921-28
AA171	ZT	Foothill	8	3918890	400976	0.0275	HRA	SAMN13420590-97
AA084	VT	Alpine	8	3447202	239213	0.0234	VEL	SAMN09759960-67
AA016	VT	Foothill	8	3915391	317662	0.0267	SUB	SAMN13420527-34

Table S3 Pearson pairwise correlation coefficients between the environmental parameters (upper) and morphological characters (lower) used in the analyses.

Ecology	PAR	Precipitation	Temperature	EIV_Moisture	EIV_Nutrients	soil_ph	EIV_Light	Veget_ cover
PAR		-0.69	0.54	-0.39	0.15	0.29	-0.15	0.14
Precipitation	-0.69		-0.65	0.20	-0.45	-0.40	0.47	-0.26
Temperature	0.54	-0.65		-0.33	0.54	0.61	-0.56	0.26
EIV_Moisture	-0.39	0.20	-0.33		0.44	-0.10	-0.28	-0.13
EIV_Nutrients	0.15	-0.45	0.54	0.44		0.44	-0.78	0.18
soil_ph	0.29	-0.40	0.61	-0.10	0.44		-0.34	0.17
EIV_Light	-0.15	0.47	-0.56	-0.28	-0.78	-0.34		-0.12
Vegetation cover	0.14	-0.26	0.26	-0.13	0.18	0.17	-0.12	

Morphology	HP	HF	PL	PW	PL.PW	SL	SW	SL.SW	SLL	SLW	SLL.SLW	RLL	RLW	RLL.RLW	SNLP	RNLP
HP		0.69	-0.21	-0.28	0.17	-0.25	-0.36	0.24	0.50	0.43	0.05	0.41	0.37	0.12	0.41	0.30
HF	0.69		-0.24	-0.26	0.11	-0.18	-0.21	0.10	0.37	0.26	0.06	0.33	0.23	0.18	0.28	0.17
PL	-0.21	-0.24		0.80	-0.03	0.72	0.55	-0.02	0.09	0.10	-0.04	0.13	0.15	-0.03	-0.03	0.01
PW	-0.28	-0.26	0.80		-0.55	0.64	0.62	-0.19	0.01	0.03	-0.07	0.06	0.09	-0.04	-0.09	-0.03
PL.PW	0.17	0.11	-0.03	-0.55		-0.07	-0.28	0.30	0.08	0.05	0.09	0.07	0.05	0.01	0.08	0.06
SL	-0.25	-0.18	0.72	0.64	-0.07		0.71	0.03	0.05	0.07	-0.04	0.13	0.15	-0.02	-0.02	0.00
SW	-0.36	-0.21	0.55	0.62	-0.28	0.71		-0.64	-0.05	-0.06	-0.06	0.03	0.03	0.00	-0.11	-0.10
SL.SW	0.24	0.10	-0.02	-0.19	0.30	0.03	-0.64		0.13	0.16	0.03	0.09	0.11	-0.02	0.14	0.16
SLL	0.50	0.37	0.09	0.01	0.08	0.05	-0.05	0.13		0.80	0.08	0.64	0.67	0.03	0.43	0.27
SLW	0.43	0.26	0.10	0.03	0.05	0.07	-0.06	0.16	0.80		-0.40	0.53	0.58	-0.02	0.56	0.29
SLL.SLW	0.05	0.06	-0.04	-0.07	0.09	-0.04	-0.06	0.03	0.08	-0.40		0.03	-0.03	0.10	-0.31	-0.05
RLL	0.41	0.33	0.13	0.06	0.07	0.13	0.03	0.09	0.64	0.53	0.03		0.77	0.39	0.25	0.26
RLW	0.37	0.23	0.15	0.09	0.05	0.15	0.03	0.11	0.67	0.58	-0.03	0.77		-0.20	0.28	0.25
RLL.RLW	0.12	0.18	-0.03	-0.04	0.01	-0.02	0.00	-0.02	0.03	-0.02	0.10	0.39	-0.20		-0.05	-0.01
SNLP	0.41	0.28	-0.03	-0.09	0.08	-0.02	-0.11	0.14	0.43	0.56	-0.31	0.25	0.28	-0.05		0.46
RNLP	0.30	0.17	0.01	-0.03	0.06	0.00	-0.10	0.16	0.27	0.29	-0.05	0.26	0.25	-0.01	0.46	

Table S4 Average values of traits in foothill and alpine groups of *A. arenosa* plants sampled in field and in common garden experiment. The measurements are provided in a Supplementary data file S3.

				Field data		Common garden data	
Traits	Description	Category	Unit	Alpine	Foothill	Alpine	Foothill
HP	Height of the main stem	stem	mm	125.05	269	114	185
HF	Distance from the rosette to the lowest flower	stem	mm	79.73	130.57	64.3	117.1
PL	Petal length	flower	mm	6.465	5.653	6.52	5.434
PW	Petal width	flower	mm	3.475	2.815	3.55	2.668
PL.PW	Ratio Petal length / width	flower	mm	1.887	2.047	1.85	2.09
SL	Sepal length	flower	mm	2.796	2.404	2.68	2.441
SW	Sepal width	flower	mm	1.428	1.093	1.14	1.067
SL.SW	Ratio Sepal length / width	flower	mm	2.014	2.264	2.41	2.332
SLL	Stem leaf length	leaf	mm	19.96	26.49	14.6	14.606
SLW	Stem leaf width	leaf	mm	5.897	7.649	4.57	5.261
SLL.SLW	Ratio Stem length / width	leaf	mm	3.721	3.943	3.35	2.944
RLL	Rosette leaf length	leaf	mm	31.65	34.36	26.6	23.401
RLW	Rosette leaf width	leaf	mm	7.385	8.647	5.58	5.405
RLL.RLW	Ratio Rosette length / width	leaf	mm	4.43	4.082	4.86	4.511
SNLP	Stem leaf number of lobe pairs	leaf lobes	count	1.865	2.585	1.6	2.914
RNLP	Rosette leaf number of lobe pairs	leaf lobes	count	3.488	4.484	4.81	6.862

Table S5 Likelihood values (MaxEstLhood$_i$ (log)), Akaike Information Criteria (AIC), delta AICs (Δ_i), Akaike weights (w_i), and parameter estimates from the best runs of each population quartet and simulated scenario. The four scenarios approximate the origin of the alpine ecotype of *A. arenosa* in pairs of mountain regions. We fully iterated alpine and foothill populations from all tetraploid regions (FG, NT, RD, and ZT) and complemented them by the spatially closest pair of diploid and tetraploid populations (ZT-VT); see Supplementary methods S3 for details. The topologies of the evaluated scenarios and the parameters are explained in Fig. S3**.**

scenario	pop. quartet	Regions	N_ params	MaxEstLhood$_i$ (log)	AIC	Δ_i	w_i	N_POP0	N_POP1	N_POP2	N_POP3	N_ANCAll	N_ANC01	N_ANC23	TDIV1	TDIV2	TDIV3
par.	BAL.DRA.INE.CAR	FG-RD	10	-278411.157	1282150.76	99.794	2.13e-22	21068	26027	44573	35294	321010	602075	626402	2174	1803	27092
par.mig	BAL.DRA.INE.CAR	FG-RD	14	-278387.75	1282050.966	0	1	9989	12927	10270	9258	332853	518754	606584	1111	459	26758
sin.	BAL.DRA.INE.CAR	FG-RD	10	-281213.849	1295057.633	13006.667	0	69685	173317	84345	159867	358202	1122729	1726361	10121	10312	10809
sin.mig	BAL.DRA.INE.CAR	FG-RD	14	-279403.338	1286727.922	4676.956	0	102428	224093	96084	193755	350127	1686713	937423	17980	17452	19787
par.	TKO.BAL.HRA.DRA	ZT-FG	10	-247190.349	1138373.625	0	1	43530	45087	11108	11748	302630	498032	604414	1520	1039	24591
par.mig	TKO.BAL.HRA.DRA	ZT-FG	14	-247210.745	1138475.553	101.928	7.36e-23	51334	66048	21407	27267	294831	483700	625217	1997	1988	28015
sin.	TKO.BAL.HRA.DRA	ZT-FG	10	-250098.955	1151768.251	13394.626	0	144521	67769	230750	77823	332889	1744297	361544	8544	10710	10831
sin.mig	TKO.BAL.HRA.DRA	ZT-FG	14	-248355.683	1143748.187	5374.562	0	170988	97034	291820	107789	299683	1327447	942946	15342	18071	18911
par.	TKO.HRA.INE.CAR	ZT-RD	10	-2719144.418	12522142.81	126.40	3.57e-28	64461	109410	35103	31306	415946	357637	529188	2874	1646	28609
par.mig	TKO.HRA.INE.CAR	ZT-RD	14	-2719115.235	12522016.41	0	1	166589	230515	28487	23229	405756	259650	533254	6886	1219	27169
sin.	TKO.HRA.INE.CAR	ZT-RD	10	-2772640.272	12768500.32	246483.91	0	120515	143021	158682	144384	440669	1305993	882176	11212	11860	11922
sin.mig	TKO.HRA.INE.CAR	ZT-RD	14	-2733993.812	12590534.79	68518.38	0	197828	241715	235571	195002	398800	256663	990436	31553	31554	32469
par.	TKO.HRA.VEL.SUB	ZT-VT	10	-1187233.085	5467430.407	11454.563	0	124391	130724	64179	92418	367550	2123732	1317191	4583	6554	7065
par.mig	TKO.HRA.VEL.SUB	ZT-VT	14	-1187363.592	5468039.414	12063.570	0	43511	46372	77688	114188	352150	2167446	1898900	1425	7728	8391
sin.	TKO.HRA.VEL.SUB	ZT-VT	10	-1184813.089	5456285.913	310.069	4.67e-68	218951	48961	226289	115115	363044	108377	348001	4598	7357	8094
sin.mig	TKO.HRA.VEL.SUB	ZT-VT	14	-1184744.021	5455975.844	0	1	261781	49654	188619	116295	362199	88170	1269569	4865	7322	7804
par.	WIL.BAL.KOS.DRA	NT-FG	10	-56927.284	262179.831	135.3359	4.09e-30	11590	20945	17107	19457	260635	452965	541965	744	1775	26649
par.mig	WIL.BAL.KOS.DRA	NT-FG	14	-56896.159	262044.4951	0	1	66650	106546	75467	72790	276583	270236	398856	3681	7548	21220
sin.	WIL.BAL.KOS.DRA	NT-FG	10	-57728.081	265867.6375	3823.1424	0	74519	66124	143462	73565	308516	1324746	641931	7862	8049	9165
sin.mig	WIL.BAL.KOS.DRA	NT-FG	14	-57301.649	263911.8456	1867.3505	0	134590	95491	200381	103154	287460	1509336	1488259	16865	15126	19561
par.	WIL.INE.KOS.CAR	NT-RD	10	-424371.665	1954323.739	0	1	13839	29491	51251	40321	365088	355131	444907	1033	1938	28586
par.mig	WIL.INE.KOS.CAR	NT-RD	14	-424419.805	1954553.432	229.693	1.32e-50	10251	21164	43262	38921	363465	426803	492232	783	2120	29114
sin.	WIL.INE.KOS.CAR	NT-RD	10	-434049.778	1998893.097	44569.358	0	80183	121514	127297	126755	404661	1587168	1396282	9753	10416	10858
sin.mig	WIL.INE.KOS.CAR	NT-RD	14	-427275.251	1967703.247	13379.508	0	138713	201894	208332	176723	357292	550659	926255	27541	28855	29434
par.	WIL.KOS.TKO.HRA	NT-ZT	10	-359355.917	1654915.155	10.651	0.00484	14948	25193	107189	172424	323802	400091	258521	909	4351	14971
par.mig	WIL.KOS.TKO.HRA	NT-ZT	14	-359351.867	1654904.504	0	0.99516	6432	13015	161918	169021	334806	536316	244664	424	5633	17638
sin.	WIL.KOS.TKO.HRA	NT-ZT	10	-363211.457	1672670.573	17766.069	0	77938	135319	121013	131520	366270	1638265	1510483	7870	7489	8148
sin.mig	WIL.KOS.TKO.HRA	NT-ZT	14	-361488.671	1664744.85	9840.346	0	127929	208094	176348	233144	309526	1531877	1463396	15941	15091	16239

Table 5 (continued)

scenario	pop. quartet	Mig01	Mig10	Mig23	Mig32	Mig02	Mig13	Mig20	Mig31
par.	BAL.DRA.INE.CAR	NA	NA	NA	NA	NA	NA	NA	NA
par.mig	BAL.DRA.INE.CAR	3.4E-06	1.8E-06	3.8E-06	6.2E-06	NA	NA	NA	NA
sin.	BAL.DRA.INE.CAR	NA	NA	NA	NA	NA	NA	NA	NA
sin.mig	BAL.DRA.INE.CAR	NA	NA	NA	NA	7.6E-06	8.1E-06	1.2E-05	1.1E-05
par.	TKO.BAL.HRA.DRA	NA	NA	NA	NA	NA	NA	NA	NA
par.mig	TKO.BAL.HRA.DRA	3E-06	5E-06	1.7E-06	1.7E-06	NA	NA	NA	NA
sin.	TKO.BAL.HRA.DRA	NA	NA	NA	NA	NA	NA	NA	NA
sin.mig	TKO.BAL.HRA.DRA	NA	NA	NA	NA	1E-05	1.2E-05	9.5E-06	9.3E-06
par.	TKO.HRA.INE.CAR	NA	NA	NA	NA	NA	NA	NA	NA
par.mig	TKO.HRA.INE.CAR	7.1E-06	4.2E-06	4.2E-06	1.6E-06	NA	NA	NA	NA
sin.	TKO.HRA.INE.CAR	NA	NA	NA	NA	NA	NA	NA	NA
sin.mig	TKO.HRA.INE.CAR	NA	NA	NA	NA	1.3E-05	1.2E-05	1.3E-05	1.3E-05
par.	TKO.HRA.VEL.SUB	NA	NA	NA	NA	NA	NA	NA	NA
par.mig	TKO.HRA.VEL.SUB	5.3E-06	1.6E-06	1.6E-06	1.2E-06	NA	NA	NA	NA
sin.	TKO.HRA.VEL.SUB	NA	NA	NA	NA	NA	NA	NA	NA
sin.mig	TKO.HRA.VEL.SUB	NA	NA	NA	NA	5.1E-06	3.3E-06	7E-06	1.5E-06
par.	WIL.BAL.KOS.DRA	NA	NA	NA	NA	NA	NA	NA	NA
par.mig	WIL.BAL.KOS.DRA	1.6E-06	3.7E-06	5.6E-06	2.5E-06	NA	NA	NA	NA
sin.	WIL.BAL.KOS.DRA	NA	NA	NA	NA	NA	NA	NA	NA
sin.mig	WIL.BAL.KOS.DRA	NA	NA	NA	NA	5.6E-06	9.1E-06	1.2E-05	8E-06
par.	WIL.INE.KOS.CAR	NA	NA	NA	NA	NA	NA	NA	NA
par.mig	WIL.INE.KOS.CAR	3.2E-06	3.8E-06	1.5E-06	2.3E-06	NA	NA	NA	NA
sin.	WIL.INE.KOS.CAR	NA	NA	NA	NA	NA	NA	NA	NA
sin.mig	WIL.INE.KOS.CAR	NA	NA	NA	NA	1.2E-05	1.3E-05	1.3E-05	1.3E-05
par.	WIL.KOS.TKO.HRA	NA	NA	NA	NA	NA	NA	NA	NA
par.mig	WIL.KOS.TKO.HRA	2E-06	1.8E-06	6.3E-06	2.4E-06	NA	NA	NA	NA
sin.	WIL.KOS.TKO.HRA	NA	NA	NA	NA	NA	NA	NA	NA
sin.mig	WIL.KOS.TKO.HRA	NA	NA	NA	NA	9.6E-06	7.9E-06	9.3E-06	1.3E-05

Table S6 Genetic and morphological diversity and differentiation of foothill and alpine populations of *A. arenosa* categorized according to four regions (treating VT and ZT regions together).

	**Genetic data**				**Morphological data from field**			**Morphological data from common garden**		
**Grouping**	**N[1]**	**AMOVA (%)[2]**	**Gen. diversity (π)[3]**	**Tajima's D**	**N[1]**	**Classif.[4]**	**Differentiation[5]**	**N[1]**	**Classif.[4]**	**Differentiation[5]**
**Grouping by region (4 regions)**										
all pops.	200	19	0.048	-0.096	999	48	6.81*** (2.01%)	223	58	5.04** (4.38%)
Foothill	109	21	0.049	-0.148	559	70	16.58*** (8.23%)	117	68	9.00*** (13.6%)
Alpine	91	28	0.048	-0.025	440	53	13.97*** (8.77%)	106	73	17.2*** (25%)
**Grouping by ecotype**										
VT+ZT	115	7	0.047 / 0.049	-0.101 / -0.244	584	88	30.41*** (4.97%)	107	94	53.7*** (33.8%)

[1] N individuals

[2] Among-group genetic variation component (in %) as explained by hierarchical AMOVA

[3] Pairwise nucleotide diversity (π) and Tajima's D values averaged over populations with ≥ 4 individuals (foothill / alpine ecotypes, respectively)

[4] % of correct classification into ecotype / regional group as inferred by classificatory discriminant analysis of the 16 morphological characters

[5] F-values and significance (*P < 0.05, **P < 0.01, ***P < 0.001) of permanova analysis of the 16 morphological characters

Table S7 Analysis of molecular variance (AMOVA) reflecting regional and ecotypic clustering of *A. arenosa* in total and within each region separately.

Grouping	dataset	SSD	MSD	% explained variance	d.f.	p
**Grouping by region (5 regions: NT, VT, ZT, RD, FG)**						
Regional group	all pops	0.0177	0.0044	19.60	4	< **0.001**
Populations within regional group	all pops	0.0359	0.0007	39.74	52	< **0.001**
Individuals within population	all pops	0.0367	0.0003	40.66	143	
Total	all pops	0.0904	0.0005	100.00	199	
Regional group	foothill pops	0.0111	0.0028	22.89	4	< **0.001**
Populations within regional group	foothill pops	0.0156	0.0007	32.10	24	< **0.001**
Individuals within population	foothill pops	0.0219	0.0003	45.01	80	
Total	foothill pops	0.0486	0.0005	100.00	108	
Regional group	alpine pops	0.0116	0.0029	29.80	4	< **0.001**
Populations within regional group	alpine pops	0.0125	0.0005	32.04	23	< **0.001**
Individuals within population	alpine pops	0.0148	0.0002	38.16	63	
Total	alpine pops	0.0389	0.0004	100.00	90	
**Grouping by region (4 regions: NT, VT+ZT, RD, FG)**						
Regional group	all pops	0.0174	0.0058	19.24	3	< **0.001**
Populations within regional group	all pops	0.0362	0.0007	40.1	53	< **0.001**
Individuals within population	all pops	0.0367	0.0003	40.66	143	
Total	all pops	0.0904	0.0005	100	199	
Regional group	foothill pops	0.0102	0.0034	**20.87**	3	< **0.001**
Populations within regional group	foothill pops	0.0166	0.0007	34.12	25	< **0.001**
Individuals within population	foothill pops	0.0219	0.0003	45.01	80	
Total	foothill pops	0.0486	0.0005	100	108	
Regional group	alpine pops	0.0108	0.0036	**27.9**	3	< **0.001**
Populations within regional group	alpine pops	0.0132	0.0005	33.94	24	< **0.001**
Individuals within population	alpine pops	0.0148	0.0002	38.16	63	
Total	alpine pops	0.0389	0.0004	100	90	
**Grouping by ecotype**						
Elevational group	all pops	0.0028	0.0028	3.12	1	< **0.001**
Populations within elevational group	all pops	0.0508	0.0009	56.22	55	< **0.001**
Individuals within population	all pops	0.0367	0.0003	40.66	143	
Total	all pops	0.0904	0.0005	100	199	
Elevational group	NT region	0.0006	0.0006	4.87	1	0.085
Populations within elevational group	NT region	0.0049	0.0005	42	10	< **0.001**
Individuals within population	NT region	0.0062	0.0002	53.13	29	
Total	NT region	0.0117	0.0003	100	40	
Elevational group	VT + ZT region	0.0036	0.0036	7.39	1	< **0.001**
Populations within elevational group	VT + ZT region	0.0156	0.0005	31.62	30	< **0.001**
Individuals within population	VT + ZT region	0.03	0.0004	60.99	83	
Total	VT + ZT region	0.0493	0.0004	100	114	
Elevational group	VT region	0.0011	0.0011	2.77	1	0.7393
Populations within elevational group	VT region	0.0178	0.001	45.86	17	< **0.001**
Individuals within population	VT region	0.0199	0.0004	51.37	52	
Total	VT region	0.0387	0.0006	100	70	

Elevational group	ZT region	0.0003	0.0003	3.39	1	0.7972
Populations within elevational group	ZT region	0.0041	0.0004	40.54	11	**< 0.001**
Individuals within population	ZT region	0.0057	0.0002	56.07	31	
Total	ZT region	0.0102	0.0002	100	43	
Elevational group	RD region	0.0004	0.0004	6.35	1	0.571
Populations within elevational group	RD region	0.002	0.0004	34.94	5	**< 0.001**
Individuals within population	RD region	0.0034	0.0002	58.71	15	
Total	RD region	0.0058	0.0003	100	21	
Elevational group	FG region	0.0006	0.0006	9.19	1	0.121
Populations within elevational group	FG region	0.0016	0.0004	24.97	4	**0.043**
Individuals within population	FG region	0.0041	0.0003	65.84	16	
Total	FG region	0.0062	0.0003	100	21	

Table S8 Pairwise differentiation (Fst) calculated based on all RAD-sequenced populations with ≥ 4 individuals.

	ecotype	region	ploidy	AA016	AA065	AA067	AA075	AA084	AA090	AA096	AA168	AA170_2	AA170_4	AA171	AA183	AA208	AA222	AA229	AA234	AA236	AA248	AA253	AA255	AA298	AA300	AA330	AA338	AA339	AA340
AA016	F	VT	2x	NA																									
AA065	A	FG	4x	0.16	NA																								
AA067	F	FG	4x	0.17	0.12	NA																							
AA075	F	RD	4x	0.15	0.14	0.13	NA																						
AA084	A	VT	2x	0.13	0.16	0.18	0.17	NA																					
AA090	A	VT	2x	0.14	0.17	0.19	0.17	0.03	NA																				
AA096	F	ZT	4x	0.07	0.14	0.15	0.14	0.1	0.1	NA																			
AA168	A	ZT	4x	0.08	0.13	0.15	0.14	0.05	0.06	0.06	NA																		
AA170_2	A	VT	2x	0.08	0.15	0.17	0.15	0.1	0.11	0.08	0.07	NA																	
AA170_4	A	ZT	4x	0.08	0.13	0.15	0.13	0.07	0.08	0.06	0.04	0.05	NA																
AA171	F	ZT	4x	0.06	0.12	0.13	0.12	0.08	0.08	0.05	0.04	0.07	0.04	NA															
AA183	F	VT	2x	0.06	0.17	0.18	0.16	0.14	0.15	0.06	0.09	0.09	0.09	0.08	NA														
AA208	F	VT	2x	0.04	0.16	0.16	0.14	0.12	0.12	0.06	0.07	0.07	0.06	0.05	0.05	NA													
AA222	A	FG	4x	0.21	0.12	0.17	0.19	0.22	0.23	0.19	0.18	0.21	0.18	0.18	0.22	0.21	NA												
AA229	F	ZT	4x	0.08	0.15	0.16	0.15	0.11	0.11	0.05	0.07	0.09	0.07	0.06	0.08	0.07	0.2	NA											
AA234	F	ZT	4x	0.08	0.15	0.16	0.14	0.09	0.1	0.04	0.06	0.08	0.06	0.05	0.08	0.07	0.2	0.05	NA										
AA236	F	VT	2x	0.08	0.2	0.21	0.18	0.16	0.16	0.09	0.12	0.11	0.11	0.1	0.09	0.07	0.25	0.11	0.1	NA									
AA248	A	VT	2x	0.13	0.18	0.2	0.18	0.04	0.05	0.09	0.07	0.11	0.09	0.09	0.14	0.12	0.24	0.09	0.08	0.16	NA								
AA253	A	NT	4x	0.18	0.2	0.2	0.19	0.18	0.2	0.15	0.15	0.17	0.15	0.14	0.19	0.17	0.25	0.16	0.16	0.22	0.2	NA							
AA255	F	NT	4x	0.15	0.17	0.18	0.16	0.15	0.16	0.13	0.12	0.14	0.12	0.11	0.16	0.14	0.22	0.13	0.13	0.18	0.17	0.12	NA						
AA298	A	RD	4x	0.17	0.14	0.14	0.07	0.18	0.19	0.15	0.15	0.17	0.15	0.13	0.18	0.16	0.2	0.16	0.16	0.2	0.2	0.2	0.17	NA					
AA300	A	NT	4x	0.12	0.14	0.15	0.14	0.13	0.14	0.1	0.09	0.12	0.09	0.09	0.14	0.12	0.2	0.11	0.11	0.16	0.14	0.09	0.08	0.15	NA				
AA330	F	FG	4x	0.14	0.1	0.06	0.11	0.15	0.16	0.13	0.12	0.14	0.12	0.11	0.16	0.14	0.15	0.14	0.13	0.18	0.17	0.18	0.15	0.12	0.13	NA			
AA338	F	NT	4x	0.13	0.15	0.15	0.13	0.15	0.15	0.11	0.11	0.13	0.11	0.1	0.15	0.13	0.2	0.12	0.12	0.17	0.16	0.14	0.11	0.15	0.09	0.13	NA		
AA339	F	NT	4x	0.16	0.17	0.18	0.16	0.16	0.17	0.14	0.13	0.15	0.13	0.12	0.17	0.15	0.23	0.15	0.14	0.19	0.18	0.16	0.13	0.17	0.1	0.15	0.12	NA	
AA340	F	NT	4x	0.12	0.15	0.15	0.14	0.13	0.14	0.1	0.1	0.12	0.1	0.09	0.13	0.12	0.2	0.11	0.1	0.16	0.14	0.11	0.08	0.15	0.06	0.13	0.09	0.1	NA

Table S9 Parallel and non-parallel morphological differentiation in 16 traits in foothill vs. alpine populations of *A. arenosa* based on field-collected samples and plants cultivated in common garden quantified using generalized linear model, calculated separately for each trait sampled in field and in common garden experiment.

		Field data						Common garden data					
		Test statistics[1]			Effect sizes[2]			Test statistics[1]			Effect sizes[2]		
Trait	Category	Ecotype[1]	Region[1]	E × R[1]	Ecotype	Region	E × R	Ecotype[1]	Region	E × R[1]	Ecotype	Region	E × R
HP	stem	151.74***	3.25*	2.02	0.48	0.06	0.05	47.06***	2.62	3.36	0.43	0.11	0.15
HF	stem	25.98***	11.90***	3.18*	0.16	0.23	0.08	54.14***	1.61	7.05*	0.52	0.08	0.30
PL	flower	10.60**	1.54	0.59	0.11	0.07	0.02	16.33**	0.77	2.25	0.27	0.06	0.13
PW	flower	23.62***	2.75*	0.33	0.20	0.09	0.01	19.43**	1.08	0.75	0.37	0.10	0.06
PL.PW	flower	21.79***	1.8	1.11	0.09	0.02	0.01	6.63*	0.91	0.22	0.16	0.07	0.02
SL	flower	17.38***	1.04	0.33	0.15	0.03	0.01	13.97**	3.75	5.26*	0.09	0.08	0.10
SW	flower	55.49***	3.40*	0.63	0.27	0.06	0.01	6.10*	1.28	0.22	0.03	0.02	0.11
SL.SW	flower	36.75***	4.52*	1.69	0.10	0.05	0.02	1.96	1.25	1.78	0.01	0.02	0.02
SLL	leaf	11.11**	0.55	4.92**	0.05	0.01	0.10	0.01	1.83	3.66	0.00	0.14	0.21
SLW	leaf	5.90*	1.01	10.95***	0.02	0.02	0.19	3.7	3.69	6.91*	0.07	0.18	0.25
SLL.SLW	leaf	1.49	0.57	6.83***	0.00	0.01	0.09	4.69	0.24	0.36	0.06	0.01	0.02
RLL	leaf	0.86	0.87	2.23	0.00	0.02	0.05	2.89	1.37	5.32*	0.04	0.06	0.20
RLW	leaf	7.79**	0.75	4.23**	0.03	0.02	0.08	0.36	0.1	3.08	0.01	0.00	0.15
RLL.RLW	leaf	5.17*	4.15**	5.02**	0.01	0.04	0.05	1.45	2.22	0.73	0.02	0.08	0.03
SNLP	leaf	27.17***	1.51	8.08***	0.10	0.03	0.12	16.53**	2.32	4.85*	0.20	0.09	0.18
RNLP	leaf	17.70***	0.76	3.65*	0.07	0.01	0.05	11.58**	1.22	4.71*	0.15	0.05	0.20

[1] F-values and corresponding significance levels in a generalized linear mixed-effect model are denoted. $*P < 0.05$, $**P < 0.01$, $***P < 0.001$. Note only the last category passes the strict Bonferroni correction.
[2] Effect sizes (Eta2 = η2) of ecotype, region and their interaction estimated using the linear model

Table S10 Morphological differentiation between foothill vs. alpine populations of *A. arenosa* quantified separately for each region using linear discriminant analysis (LDA). The analysis was based on in 16 traits collected in field samples and plants cultivated in common garden.

		Field data						Common garden data				
Trait	Category	All data	NT	VT	ZT	RD	FG	All data	NT	VT	ZT	FG
HP	stem	**0.74**	**-0.36**	**0.33**	**0.45**	**-0.61**	**0.78**	**-0.59**	**-0.39**	**-0.48**	**0.28**	**0.38**
HF	stem	**0.40**	0.02	**0.43**	**0.49**	**-0.42**	0.16	**-0.62**	**-0.39**	**-0.61**	**0.22**	**0.58**
PL	flower	**-0.28**	0.07	**-0.30**	-0.20	**0.41**	-0.10	**0.44**	**0.66**	**0.47**	-0.18	-0.07
PW	flower	**-0.40**	0.13	**0.39**	**0.48**	**0.35**	-0.18	**0.56**	**0.79**	**0.47**	**-0.30**	-0.16
PL.PW	flower	**0.26**	-0.10	**0.44**	**0.64**	0.03	0.14	**-0.32**	**-0.49**	-0.09	**0.22**	0.19
SL	flower	**-0.36**	0.12	-0.20	**-0.30**	0.16	-0.12	0.23	0.16	**0.37**	-0.11	-0.01
SW	flower	**-0.52**	0.20	**-0.60**	**-0.70**	0.21	**-0.33**	0.12	-0.07	0.25	-0.14	0.05
SL.SW	flower	**0.27**	-0.13	**-0.60**	**-0.60**	-0.11	**0.27**	0.07	0.20	0.01	0.07	-0.08
SLL	leaf	0.23	0.01	**-0.30**	**-0.30**	0.01	**0.43**	0.01	0.02	0.21	-0.14	0.25
SLW	leaf	0.18	0.02	**-0.40**	-0.01	0.17	**0.35**	-0.16	**-0.28**	0.09	-0.09	**0.38**
SLL.SLW	leaf	0.06	-0.04	0.01	-0.10	**-0.32**	-0.07	0.21	**0.29**	0.06	-0.06	-0.16
RLL	leaf	0.08	0.02	-0.01	-0.20	-0.16	**0.28**	0.14	-0.01	0.03	**-0.50**	0.09
RLW	leaf	0.16	-0.05	-0.10	-0.20	-0.03	**0.34**	0.05	-0.20	0.05	**-0.40**	0.14
RLL.RLW	leaf	-0.10	0.10	-0.10	0.03	-0.25	-0.06	0.10	0.10	-0.01	-0.21	-0.01
SNLP	leaf	**0.27**	-0.01	0.05	-0.10	0.06	**0.40**	**-0.33**	-0.15	-0.17	0.04	**0.64**
RNLP	leaf	**0.25**	-0.04	0.08	-0.01	0.04	0.22	**-0.31**	-0.05	-0.24	0.03	**0.33**

Loadings on the first (constrained) axis of linear discriminant analysis (LDA) between foothill and alpine individuals calculated for the complete dataset ('all') and each region separately based on all 16 scored traits. The traits with moderate to high contribution to the discrimination (> 0.25 absolute value of the loading coefficient) are highlighted in bold.

Supplementary data file 1 Observed allele frequency spectra, (*.*dsfs* files), estimated *.*par* files for best likelihood runs and corresponding input parameter files (*.*est* and *.*tpl* files) for all scenarios addressed by coalescent simulations in fastsimcoal

Supplementary data file 2 Vegetation samples and environmental parameters sampled at original sites of *A. arenosa* populations

Supplementary data file 3 Morphological traits of samples from field (sheet 1) and common garden (sheet 2).

Supplementary data file 4 R-scripts used for analyses of the variation in morphological traits.
