# Supplementary material for: Parallel Alpine Differentiation in Arabidopsis arenosa
Source: Front Plant Sci. 2020 Dec 8;11:561526. doi: 10.3389/fpls.2020.561526 (PMC7753741; doi:10.3389/fpls.2020.561526)
Supplement: Supplementary file 5 [file Data_Sheet_5.docx]

# Used with MorphoTools

# Koutecký P. (2015): MorphoTools: a set of R functions for morphometric analysis. - Plant Systematics and Evolution 301: 1115-1121, DOI 10.1007/s00606-014-1153-2

# DATA IMPORT AND CONTROL

data.orig<-read.morphodata("source_file.txt")

# substitutes NAs with pop. means

data<-na.meansubst(data.orig)

# based on the char. distribution we log10 these chars

data$morphological_character<-log10(data$morphological_character)

# PCA of individuals

indivpca<-pca.calc(data)

summary(indivpca)

pca.eigen(indivpca) # eigenvalues

plot(indivpca) # scree plot

plot(indivpca,type="lines") # scree plot

indivpca.cor<-pca.cor(indivpca) # loadings of the characters

indivpca.scores<-pca.scores(indivpca,data) # sample scores

# CANONICAL DISCRIMINANT ANALYSIS

# using the package vegan

# for disriminant analysis, binary characters invariable in some group should be removed

# by column numbers:

## discriminant analysis of individuals

indiv_dis<-discr.calc(data)

discr.sum(indiv_dis, perm=1000)

indiv.coef<-discr.coef(indiv_dis)

export.res(indiv.coef)

indiv.taxa<-discr.taxa(indiv_dis)

export.res(indiv.taxa)

indiv.scores<-discr.scores(indiv_dis,data)

export.res(indiv.scores)

indiv.bip<-discr.bip(indiv_dis)

export.res(indiv.bip)

indiv.test<-discr.test(indiv_dis)

indiv.test

export.res(indiv.test[[1]])

export.res(indiv.test[[2]])

discr.step(indiv)

# CLASSIFICATORY DISCRIMINANT ANALYSIS

# ID as leavout unit

classif_id<-classif.da(data,crossval="ID")

classif_id_matrix<-classif.matrix(classif_id)

classif_id_pmatrix<-classif.pmatrix(classif_id)

# POP as leavout unit

classif_pop<-classif.da(data,crossval="pop")

classif_pop_matrix<-classif.matrix(classif_pop)

classif_pop_pmatrix<-classif.pmatrix(classif_pop)

#DISPARITY

library(dispRity)

species <- data[,3]

species

unique(species)

ordination <- pca.calc(data)

disp_space <- ordination$x

## Adding the elements names to the petal-space (the individuals IDs)

rownames(disp_space) <- 1:nrow(disp_space)

## Creating the table that contain the elements and their attributes

disp_subsets <- custom.subsets(disp_space, group = list(

"X" = which(species == "X"),

"Y" = which(species == "Y")))

disp_subsets

(disp_bootstrapped <- boot.matrix(disp_subsets, bootstraps = 1000))

## Calculating disparity as the median distance between each elements and

## the centroid of the petal-space

(morfo_disparity <- dispRity(disp_bootstrapped, metric = c(median, centroids)))

summary(morfo_disparity)

## Running a PERMANOVA

test.dispRity(morfo_disparity, test = adonis.dispRity)

## Post-hoc testing of the differences between species (corrected for multiple tests)

test.dispRity(morfo_disparity, test = t.test, correction = "bonferroni")
